# Tuning Excited State Character in Iridium(III) Photosensitizers and Its Influence on TTA-UC

**DOI:** 10.1021/acs.inorgchem.4c01003

**Published:** 2024-05-13

**Authors:** Ibrahim S. Alkhaibari, Xue Zhang, Jianzhang Zhao, Thomas M. Stonelake, Richard C. Knighton, Peter N. Horton, Simon J. Coles, Niklaas J. Buurma, Emma Richards, Simon J. A. Pope

**Affiliations:** † School of Chemistry, Main Building, Cardiff University, Cardiff, Cymru/Wales CF10 3AT, U.K.; ‡ Department of Chemistry, College of Science, Qassim University, Buraydah 52571, Saudi Arabia; § State Key Laboratory of Fine Chemicals, Frontiers Science Center for Smart Materials, School of Chemical Engineering, Dalian University of Technology, Dalian 116024, PR China; ∥ School of Chemistry, University of Southampton, Highfield, Southampton SO17 1BJ, U.K.; ⊥ UK National Crystallographic Service, Chemistry, Faculty of Natural and Environmental Sciences, University of Southampton, Highfield, Southampton SO17 1BJ, U.K.

## Abstract

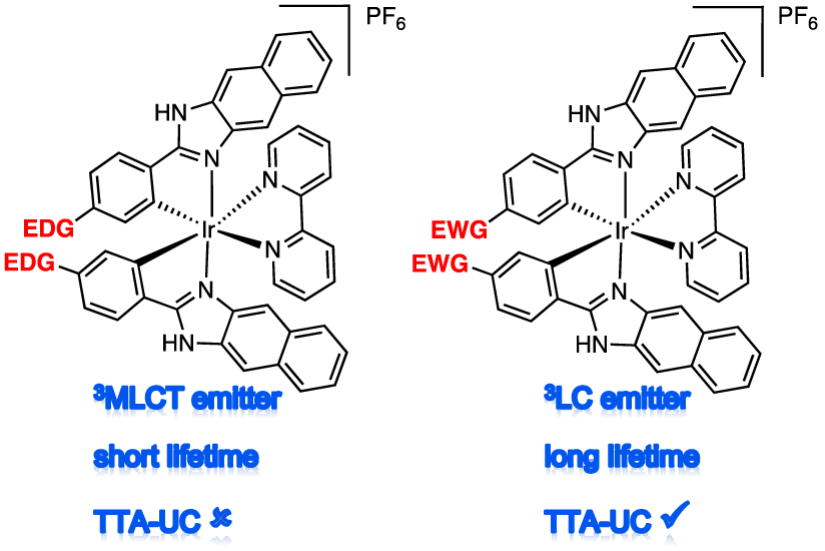

A series of mixed ligand, photoluminescent organometallic Ir(III) complexes have been synthesized to incorporate substituted 2-phenyl-1*H*-naphtho[2,3-*d*]imidazole cyclometalating ligands. The structures of three example complexes were categorically confirmed using X-ray crystallography each sharing very similar structural traits including evidence of interligand hydrogen bond contacts that account for the shielding effects observed in the ^1^H NMR spectra. The structural iterations of the cyclometalated ligand provide tuning of the principal electronic transitions that determine the visible absorption and emission properties of the complexes: emission can be tuned in the visible region between 550 and 610 nm and with triplet lifetimes up to 10 μs. The nature of the emitting state varies across the series of complexes, with different admixtures of ligand-centered and metal-to-ligand charge transfer triplet levels evident. Finally, the use of the complexes as photosensitizers in triplet–triplet annihilation energy upconversion (TTA-UC) was investigated in the solution state. The study showed that the complexes possessing the longest triplet lifetimes showed good viability as photosensitizers in TTA-UC. Therefore, the use of an electron-withdrawing group on the 2-phenyl-1*H*-naphtho[2,3-*d*]imidazole ligand framework can be used to rationally promote TTA-UC using this class of complex.

## Abstract 2

Simple variation of a cyclometalated ligand substituent results in the defined switching of the triplet excited state within a series of Ir(III) complexes. The longest-lived triplet emitters show viability as photosensitizers for triplet−triplet annihilation energy upconversion.

## Introduction

Energy upconversion, which converts low-energy photons into high-energy photons, is a photophysical phenomenon that has important applications in, and implications for, several critical technological areas. These include light harvesting and solar materials, photocatalysis, bioimaging, security, and communications technologies. One of our ongoing interests is the development of light-activated, sensitizer molecules that can be used in triplet–triplet annihilation energy upconversion (TTA-UC).^1^ TTA-UC relies upon two photoactive molecular components, a sensitizer and an annihilator. Light absorption is therefore dictated by the sensitizer species and attractive properties for maximizing TTA-UC efficiency include good molar absorption and a long triplet excited state lifetime. The triplet level (T_1_) of the annihilator species, commonly a polyaromatic molecule such as 9,10-diphenylanthracene (DPA), must allow forward triplet–triplet energy transfer (TTET) from a compatible photosensitizer. Following annihilation, upconverted delayed fluorescence then occurs from the S_1_ level of the annihilator molecule.

Photoactive transition metal complexes have proven very attractive candidates as photosensitizers for TTA-UC, as they can marry good molar absorption in the visible region with extended triplet excited state lifetimes. Ru(II),^2^ Re(I),^3^ and Ir(III)^4^ complexes have been successfully utilized to produce TTA-UC efficiencies up to 39.3%.^5^ Pt(II) species have also been investigated in TTA-UC,^6^ including organometallic complexes^7^ and Schiff base ligand complexes^8^ and the well-known Pt(II) octaethylporphyrin,^9^ which combines very efficient visible light absorption with a microsecond duration lifetime of the triplet state.^10,11^ Interestingly, a phosphorescent Cr(III) complex has recently been reported in TTA-UC, demonstrating an alternative sensitizing mechanism utilizing Cr-centered excited states.^12^

In this context, perhaps the most valuable attribute of photoactive organometallic Ir(III) complexes is the ability to finely tune the excited triplet state properties, especially through different ligand combinations^13^ and ligand substituents:^14^ there is a substantial body of work based on a detailed understanding of the principles that underpin color tuning in such systems, including on the nature of the lowest excited triplet state,^15^ which is essential for applications such as light-emitting devices.^16^ In TTA-UC, the importance of this has been demonstrated through studies which show that even very subtle changes to the excited state properties (achieved through relatively trivial changes in ligand structure) can result in profound variations in TTA-UC behavior.^5,17^

Building on these advances, in this paper, we investigated the TTA-UC characteristics of a series of structurally analogous cyclometalated Ir(III) complexes. This study was underpinned by a detailed description of the photophysical properties of the complexes, which reveal how a single point of functional variation on a chelating ligand can result in a fundamental change in triplet excited state character and thus modulation of the resultant TTA-UC behavior. The study reveals that for a given ligand system, structural changes can be rationally invoked to optimize Ir(III) photosensitizers for TTA-UC.

## Experimental Section

^1^H, ^19^F{^1^H}, and ^13^C{^1^H} NMR spectra were recorded in d6-DMSO on an NMR-FT Bruker 400 or 500 MHz spectrometer. ^1^H and ^13^C{^1^H} NMR chemical shifts (δ) were determined relative to residual solvent peaks with digital locking and are given in ppm. Coupling constants are quoted in Hz. Mass spectra were obtained by the staff at Cardiff University. The UV/vis absorption spectra were recorded with the UV2550 spectrophotometer (Shimadzu Ltd., Japan). The emission spectra were recorded with an FS5 spectrofluorometer (Edinburgh Instruments, UK). The experimental lifetimes were recorded with an OB920 luminescence lifetime spectrometer (Edinburgh Instruments, UK), and time-correlated single photon counting (TCSPC) method was used. Quantum yield measurements were obtained on aerated MeCN solutions of the complexes using [Ru(bipy)_3_](PF_6_)_2_ in aerated MeCN as a standard (Φ = 0.018).^18^

The nanosecond transient absorption spectra were acquired on LP980 laser flash photolysis spectrometer (Edinburgh Instruments, UK). The signal was digitized with a Tektronix TDS 3012B oscilloscope. The samples were excited with a nanosecond pulsed laser (Surelite I-10, USA; the wavelength is tunable in the range of 410–2400 nm). The typical laser energy is 5 mJ per pulse. The samples were deaerated with N_2_ for 15 min prior to investigations. The data were processed by L900 software.

## Cyclic Voltammetry

Cyclic voltammetry was conducted employing a PalmSens4 potentiostat. HPLC grade MeCN served as the solvent with an analyte concentration of 1 mM at 293 K, utilizing triply recrystallized [^*n*^Bu_4_N][PF_6_] as the supporting electrolyte at a concentration of 0.1 M. The experimental setup included a three-electrode system comprising a platinum disc working electrode, a platinum wire counter-electrode, and a silver wire pseudoreference electrode. Prior to experimentation, solutions were purged for 10 min with a MeCN-saturated stream of nitrogen gas. Voltammograms were referenced to the ferrocene/ferrocenium redox couple measured under the same conditions.

## X-Ray Crystallography

### Data Collection and Processing

Suitable crystals of **Ir–OMe**, **Ir–H**, and **Ir–Me** were selected and data collected following a standard method.^19^ For each compound, the selected crystal was mounted on a MITIGEN holder in oil on a Rigaku FRE+ diffractometer with Arc)Sec VHF Varimax confocal mirrors, a UG2 goniometer, and HyPix 6000HE detector. Each crystal was kept at a steady *T* = 100(2) K during data collection. The structures were solved with the ShelXT^20^ structure solution program using the intrinsic phasing solution method and by using Olex2^21^ as the graphical interface. The model was refined with version 2018/3 of ShelXL^22^ using least squares minimization. CCDC 2336520–2336522 contains supplementary X-ray crystallographic data for XXXX. This data can be obtained free of charge via http://www.ccdc.cam.ac.uk/conts/retrieving.html, or from the Cambridge Crystallographic Data Centre, Union Road, Cambridge, CB2 1EZ; fax(+44) 1223-336-033 or email: deposit@ccdc.cam.ac.uk.

### Computational Methods

Electronic structure calculations were all performed using density fitted-density functional theory within the Gaussian 09 computational chemistry suite.^23^ All calculations were performed using the Stuttgart-Dresden (SDD) effective core potential and basis set in the treatment of the iridium,^24^ in combination with a 6-31G* basis set for all other light atoms.^25^ Full geometry optimizations were performed for the cationic complexes utilizing the self-consistent reaction field (SCRF) modelwhich treats the solvent implicitly as a dielectric continuum. In all cases, the solvent chosen was dichloromethane.

All geometry optimizations were performed using an ultrafine grid and very tight convergence criteria, and the minima were confirmed as stationary points through the computation of harmonic vibrational frequencies, each of which showed no imaginary components. These stationary points were used in single point TD-DFT calculations to compute vertical excitation energies. All TD-DFT calculations were undertaken using a linear response approach. All TD-DFT calculations were also performed with a long-range corrected hybrid functional (CAM-B3LYP).

Phosphorescence and spin-forbidden absorption bands were investigated using unrestricted density functional theory to compute parameters associated with the first triplet state (T_1_), using an identical methodology as for the singlet states. Decomposition of the molecular orbital character was performed using the GaussSum software package.^26^ Crystal structure overlays with optimized computational structures has been performed using the Chimera software package, which has also been used to calculate root mean squared deviation (RMSD) values for these comparative structures.^27^

## Synthesis

### General Procedure for Iridium(III) Complexes Synthesis

IrCl_3_·*x*H_2_O (assumed trihydrate) (1.0 equiv) and imidazole-naphthalene ligand (2.0 equiv) were added to a Schlenk flask under N_2_ with the addition of 2-ethoxyethanol/H_2_O (3:1). The reaction was heated to reflux for 74 h, then cooled, and water was added. After filtering the reaction mixture, the crude solid was washed with water and dried under vacuum to obtain the iridium dimer which was then used without further purification. The iridium(III) dimer (1.0 equiv) and 2,2′-bipyridine (2.0 equiv) were added to a Schlenk flask along with 2-ethoxyethanol under a N_2_ atmosphere and heated for 36 h. The reaction was then cooled and 0.1 M NH_4_PF_6_ was added. The resultant precipitate was filtered, rinsed with water, and dried in vacuo. Purification of the crude product was achieved using column chromatography (SiO_2_; DCM/MeOH; solvent gradient 100:0 → 90:10).

### [Ir(L1)_2_(bipy)][PF_6_]

Isolated as an orange powder; (280 mg, 87%) ^1^H NMR (500 MHz, d6-DMSO) δ: 14.96 (br, 2H, NH), 8.93 (d, *J*_HH_ = 8.4 Hz, 2H), 8.38 (app. t, *J*_HH_ = 8.3 Hz, 2H), 8.30–8.25 (m, 4H), 8.12 (s, 2H), 8.02 (d, *J*_HH_ = 8.6 Hz, 2H), 7.85 (app. t, *J*_HH_ = 6.6 Hz, 2H), 7.38 (app. t, *J*_HH_ = 8.0 Hz, 2H), 7.30 (app. t, *J*_HH_ = 8.0 Hz, 2H), 7.23 (d, *J*_HH_ = 8.0 Hz, 2H), 7.05 (app. t, *J*_HH_ = 7.6 Hz, 2H), 6.84 (t, *J*_HH_ = 7.2 Hz, 2H), 6.36 (d, *J*_HH_ = 7.7 Hz, 2H), 5.90 (s, 2H) ppm; ^13^C{^1^H} NMR (126 MHz, d6-DMSO) δ: 168.4, 156.7, 152.1, 151.3, 139.5–139.4, 134.0–133.8, 132.7, 131.1, 129.8–129.6, 128.4, 127.7, 127.2, 125.9, 124.5–124.3, 122.0, 109.1–108.6 ppm; HR MS (ES+): *m*/*z* calcd 835.22 for C_44_H_30_IrN_6_; found 835.2177. IR (ATR, cm^–1^): 3387 (NH), 1593 (C=N), 1529 (C=C), 1467 (C–N), 1446 (C–C).

### [Ir(L2)_2_(bipy)][PF_6_]

Isolated as a red powder; (432 mg, 87%) ^1^H NMR (500 MHz, d6-DMSO) δ: 14.73 (br, 2H, NH), 8.88 (d, *J*_HH_ = 8.5 Hz, 2H), 8.35 (td, *J*_HH_ = 1.8, 8.3 Hz, 2H), 8.28 (d, *J*_HH_ = 7.5 Hz, 2H), 8.14 (d, *J*_HH_ = 7.8 Hz, 2H), 8.09 (s, 2H), 8.00 (d, *J*_HH_ = 8.3 Hz, 2H), 7.84 (app. t, *J*_HH_ = 6.7 Hz, 2H), 7.36 (app. t, *J*_HH_ = 7.6 Hz, 2H), 7.28 (app. t, *J*_HH_ = 7.7 Hz, 2H), 7.21 (d, *J*_HH_ = 7.6 Hz, 2H), 6.88 (d, *J*_HH_ = 6.5 Hz, 2H), 6.20 (s, 2H), 5.83 (s, 2H), 1.98 (s, 6H, CH_3_) ppm; ^13^C{^1^H} NMR (126 MHz, d6-DMSO) δ: 168.4, 156.7, 152.4, 151.3, 140.9, 139.5–139.4, 133.7–133.4, 131.3, 129.8–129.7, 128.5, 127.7, 127.2, 125.9, 124.5–124.4, 123.3, 108.9–108.4, 21.8 (CH_3_) ppm; HR MS (ES+): *m*/*z* calcd 863.25 for C_46_H_34_IrN_6_; found 863.2480. IR (ATR, cm^–1^): 3383 (NH), 2964 (C–H), 1591 (C=N), 1525 (C=C), 1500 (C–N), 1444(C–C).

### [Ir(L3)_2_(bipy)][PF_6_]

Isolated as a brown powder; (411 mg, 96%) ^1^H NMR (500 MHz, d6-DMSO) δ: 13.87 (br, 2H, NH), 8.90 (d, *J*_HH_ = 8.2 Hz, 2H), 8.43–8.33 (m, 4H), 8.03 (app. t, *J*_HH_ = 8.0 Hz, 6H), 7.88 (app. t, *J*_HH_ = 6.3 Hz, 2H), 7.35–7.29 (m, 4H), 7.23 (d, *J*_HH_ = 7.2 Hz, 2H), 6.75 (dd, *J*_HH_ = 2.2, 6.1 Hz, 2H), 5.85 (s, 2H), 5.77–5.76 (m, 2H), 3.47 (s, 6H, CH_3_) ppm; ^13^C{^1^H} NMR (126 MHz, d6-DMSO) δ: 168.1, 161.4, 156.7, 154.1, 151.5, 139.7–139.5, 133.7, 129.8–129.8, 128.6, 127.7, 127.4–127.3, 126.6, 124.6–124.5, 119.1, 108.7–108.4, 106.8, 54.6 ppm; HR MS (ES+): *m*/*z* calcd 895.24 for C_46_H_34_IrN_6_O_2_; found 895.2396. IR (ATR, cm^–1^): 3392 (NH), 1597 (C=N), 1589 (C=C), 1444 (C–N), 1425 (C–C), 1230 (C–O).

### [Ir(L4)_2_(bipy)][PF_6_]

Isolated as a yellow beige powder; (399 mg, 92%) ^1^H NMR (500 MHz, d6-DMSO) δ: 14.51 (br, 2H, NH), 8.91 (d, *J*_HH_ = 8.5 Hz, 2H), 8.42 (td, *J*_HH_ = 1.7, 7.8 Hz, 2H), 8.32 (d, *J*_HH_ = 7.1 Hz, 2H), 8.17 (s, 3H), 8.16 (s, 1H), 8.05 (d, *J*_HH_ = 7.9 Hz, 2H), 7.89 (qd, *J*_HH_ = 1.3, 5.4 Hz, 2H), 7.42 (app. t, *J*_HH_ = 6.5 Hz, 2H), 7.34 (app. t, *J*_HH_ = 6.6 Hz, 2H), 7.26 (d, *J*_HH_ = 7.8 Hz, 2H), 7.22 (dd, *J*_HH_ = 2.0, 6.2 Hz, 2H), 6.22 (d, *J*_HH_ = 2.0 Hz, 2H), 5.89 (s, 2H) ppm; ^13^C{^1^H} NMR (126 MHz, d6-DMSO) δ: 167.1, 156.5, 153.6, 151.7, 140.0, 138.9, 135.9, 133.3–132.8, 131.8, 130.0–129.8, 128.8, 127.8, 127.3, 124.7, 122.7, 109.5–108.9 ppm; HR MS (ES+): *m*/*z* calcd 903.14 for C_44_H_28_Cl_2_IrN_6_; found 903.1366. IR (ATR, cm^–1^): 3433 (NH), 1583 (C=N), 1527 (C=C), 1444 (C–N), 1417 (C–C), 1271 (C–O).

### [Ir(L5)_2_(bipy)][PF_6_]

Isolated as a yellow powder; (516 mg, 93%) ^1^H NMR (500 MHz, d6-DMSO) δ: 14.51 (br, 2H, NH), 8.94 (d, *J*_HH_ = 8.4 Hz, 2H), 8.39 (td, *J*_HH_ = 1.6, 7.9 Hz, 2H), 8.27–8.25 (m, 6H), 8.08 (d, *J*_HH_ = 8.5 Hz, 2H), 7.89 (dt, *J*_HH_ = 1.5, 7.0 Hz, 2H), 7.50–7.44 (m, 4H), 7.86 (td, *J*_HH_ = 1.2, 6.8 Hz, 2H), 7.30 (d, *J*_HH_ = 7.6 Hz, 2H), 6.51 (s, 2H), 5.96 (s, 2H) ppm; ^13^C{^1^H} NMR (126 MHz, d6-DMSO) δ: 166.8, 156.5, 151.7 (q, ^2^*J*_C–F_ = 20.0 Hz), 139.0 (q, ^1^*J*_C–F_ = 262.8 Hz), 138.8, 133.2, 130.2, 129.9, 128.9, 127.8, 127.4, 125.8, 124.9 (q, ^3^*J*_C–F_ = 6.2 Hz), 124.7, 122.5, 119.7, 109.9–109.2 ppm; ^19^F{^1^H} NMR (471 MHz, d6-DMSO) δ: −61.16 (s, CF_3_) ppm; HR MS (ES+): *m*/*z* calcd 971.19 for C_46_H_28_F_6_IrN_6_; found 971.1912. IR (ATR, cm^–1^): 3468 (NH), 1602 (C=N), 1527 (C=C), 1481 (C–N), 1446 (C–C), 1311 (C–F).

## Results and Discussion

### Synthesis of the Ligands and Complexes

**Scheme 1:**
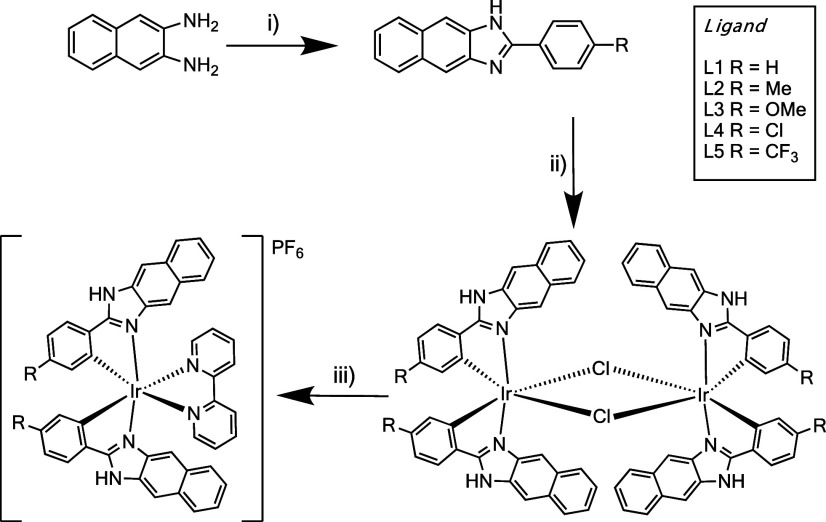
General Synthetic Scheme for the Ligands and Complexes Reagents and conditions: (i) Benzaldehyde EtOH, NH_4_Cl, or Na_2_S_2_O_3_, reflux; (ii) 0.5 eq. IrCl_3_·*x*H_2_O, 2-ethoxyethanol, water, heat; (iii) 2 eq. 2,2′-bipyridine, 2-ethoxyethanol, heat, NH_4_PF_6_.

In this study, we were interested in a conjugated cyclometalating ligand architecture, with ease of functionality, which could promote triplet excited states of both ligand-centered (LC) and metal-to-ligand charge transfer (MLCT) character in heteroleptic cationic Ir(III) species. The overall synthetic pathway to the complexes, via the ligands, is therefore shown in Scheme 1. The utilized ligands (**L1–L5**), based on a 2-phenyl-1*H*-naphtho[2,3-*d*]imidazole structural core, were achieved in a simple one-step condensation reaction between 2,3-diaminonaphthalene and the relevant benzaldehyde as noted previously.^22−24^ The different benzaldehydes conveniently introduce a range of substituents of differing electronic classifications to the phenyl component of the ligand. The formation of the ligands was confirmed using ^1^H NMR spectroscopic studies with the signature N*H* resonance noted at around 12.8 ppm; this resonance was subtly shifted downfield for the electron-withdrawing Cl and CF_3_ substituted variants. For completion and reference, all relevant spectral data for **L1–L5** are presented in Figures S1–S10. This series of ligands was then utilized as cyclometalating agents for Ir(III) using the traditional approach described by Nonoyama^28^ to yield, first, the μ-dichloro bridged dimer species, [(C^N)_2_Ir-μ-Cl_2_–Ir(C^N)_2_]. Subsequent addition of 2,2′-bipyridine in 2-ethoxyethanol and anion exchange using aqueous NH_4_PF_6_ yielded the crude monometallic cationic complexes, [Ir(**L**)_2_(bipy)]PF_6_. Further purification using column chromatography gave the five complexes as colored, air-stable solids.

**Scheme 2:**
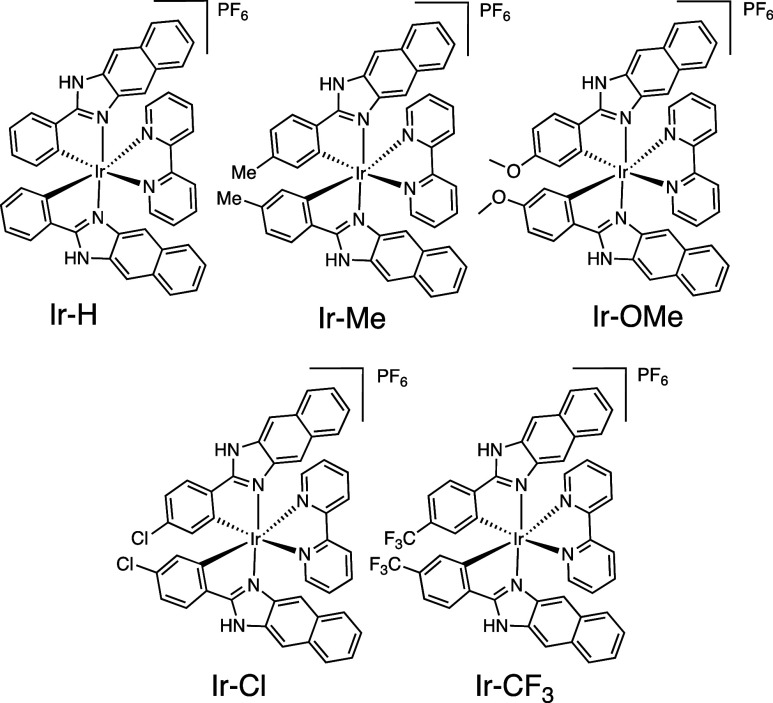
Family of Cyclometalated Iridium(III) Complexes Investigated in This Study

The proposed molecular structures of the isolated complexes investigated in this study are shown in Scheme 2. Again, ^1^H NMR spectroscopy helped provide categorical evidence for the formation of the desired species. First, the two C^N ligands are equivalent and present one set of ^1^H resonances which were easily resolved. In all cases, the N*H* signal was shifted downfield to 14–15 ppm, indicative of coordination to Ir(III). Both **Ir–Me** and **Ir–OMe** possess an additional aliphatic signal associated with the ligand substituent; these were noted at 1.98 and 3.47 ppm, respectively, representing an upfield shift in each case (cf 2.40 and 3.86 ppm for **L2** and **L3**) induced by coordination to the iridium center. A proton resonance associated with the 3-position of the naphthyl ring was observed as a singlet (integral of 2H) at ca. 5.8–5.9 ppm. This highly shielded aromatic resonance is attributed to the orientation and spatial proximity of this proton to the aromatic ring of the coordinated 2,2′-bipyridine ligand (see later discussion of the X-ray crystal structure details) as noted in other benzimidazole systems.^29 13^ C{^1^H} NMR data was also obtained for each complex and revealed a furthest downfield resonance ca. 168 ppm, assigned to the (naphthyl) imidazolyl carbon. The anticipated C–F coupling arising from the trifluoromethane group in **Ir–CF**_**3**_ was identified via both ^1^*J*_CF_ and ^3^*J*_CF_ in aromatic resonances between 120 and 140 ppm. For **Ir–CF**_**3**_, ^19^F{^1^H} NMR spectroscopy revealed a singlet at −61.16 ppm, which was consistent with an aryl CF_3_ substituent,^30^ and coincidentally close to the value of the free ligand, **L5** (−61.29 ppm). All spectra are available in Figures S10–S21.

IR spectroscopy data for the complexes showed a NH vibrational stretch around 3392–3433 cm^–1^, indicating that the NH group is retained in the complexes, consistent with the proposed binding mode (i.e. the ligand is not dianionic). The Ir(III) complexes gave excellent HRMS data (Figure S22) with *m*/*z* values that were consistent with the cationic complex fragment in each case.

### Single-Crystal X-Ray Crystallographic Studies of Ir–OMe, Ir–H, and Ir–Me

**Figure 1:**
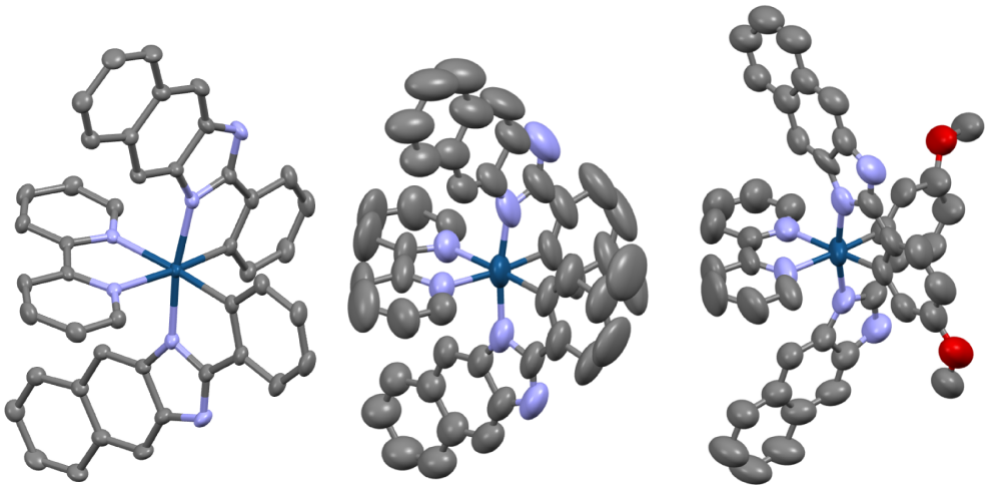
Structures obtained from the single crystal X-ray diffraction studies of (from left to right) **Ir–H**, **Ir–Me**, and **Ir–OMe** (H-atoms, solvent molecules and counteranions are omitted for clarity). Ellipsoids drawn at 50% probability.

Suitable crystals were obtained for X-ray diffraction studies on three of the complexes using either vapor diffusion of ^*i*^Pr_2_O into concentrated solutions of the complex in MeCN (**Ir–OMe** and **Ir–Me**) or a mixture of methanol and hexane (**Ir–H**). The data collection parameters are shown in Table S1. In summary, the diffraction data confirmed the proposed formulations and geometries for the complexes (Figure 1). The 2-phenyl-1*H*-naphtho[2,3-*d*]imidazole cyclometalating ligands coordinate in the expected fashion with a mutually *cis*-C,C arrangement to the Ir–C bonds, inducing a modestly distorted octahedral geometry. There is very little intraligand distortion noted for the conjugated ligands. The principal bond lengths (Table 1; for bond angles, see Table S2) that describe the coordination spheres are consistent with a recent report on related complexes,^31^ as well as earlier work on chromophore conjugated benzimidazole derivatives.^32^ Each of the structures shows that the 3-position naphthyl ring proton is oriented toward the plane of the 2,2′-bipyridine ligand. The resultant H – centroid distances are 2.6363(12) and 2.9334(14) Å (for **Ir–H**), 2.955(5) and 2.896(5) Å (for **Ir–Me**), and 2.886(6), 2.847(6), and 3.122(6) Å (for **Ir–OMe**) which may help explain the pronounced shielding observed for this proton in the ^1^H NMR spectra of the complexes.

**Table 1:** Iridium ligand Bond Lengths (Å) for the Three Crystal Structures

	bond lengths (Å)			
**Ir–H**	Ir1–N1′	2.033(2)	Ir1–N41	2.118(2)
	Ir1–N1	2.033(2)	Ir1–C1′	2.026(2)
	Ir1–N41′	2.118(2)	Ir1–C1	2.026(2)
	Ir2–N21′	2.038(2)	Ir2–N51	2.119(2)
	Ir2–N21	2.038(2)	Ir2–C21′	2.024(3)
	Ir2–N51′	2.119(2)	Ir2–C21	2.024(3)
**Ir–Me**	Ir1–N1	2.032(8)	Ir1–N42	2.132(7)
	Ir1–N21	2.010(9)	Ir1–C1	2.003(9)
	Ir1–N41	2.105(7)	Ir1–C21	2.030(10)
**Ir–OMe**	Ir1–N1	2.041(7)	Ir1–N42	2.172(8)
	Ir1–N21	2.052(8)	Ir1–C1	2.002(9)
	Ir1–N41	2.129(8)	Ir1–C21	2.024(11)
	Ir1–N21b	2.073(15)	Ir1–C21b	2.023(18)

### Spectroscopic, Redox, and Electronic Properties

The electrochemical properties of the series of iridium complexes were explored using cyclic voltammetry in degassed MeCN solution at a 1 mM concentration, using [^*n*^Bu_4_N][PF_6_] as the supporting electrolyte (0.1 M) and the Fc/Fc^+^ redox couple as a reference. The complexes generally showed very poor electrochemical stability (Figure S23), but each complex showed an irreversible oxidation above +1.0 V which was tentatively assigned to the Ir^3+/4+^ couple. The complexes with electron-withdrawing substituents, **Ir–Cl** and **Ir–CF**_**3**_, appeared to be the hardest to oxidize which is consistent with a metal-based process. Both **Ir–Cl** and **Ir–CF**_**3**_ also showed an additional irreversible feature ca. + 0.5 V which is tentatively assigned to oxidation of the secondary amine group within the ligand.^33^ Each complex showed several reduction features which were typically irreversible suggesting poor electrochemical stability.

**Figure 2:**
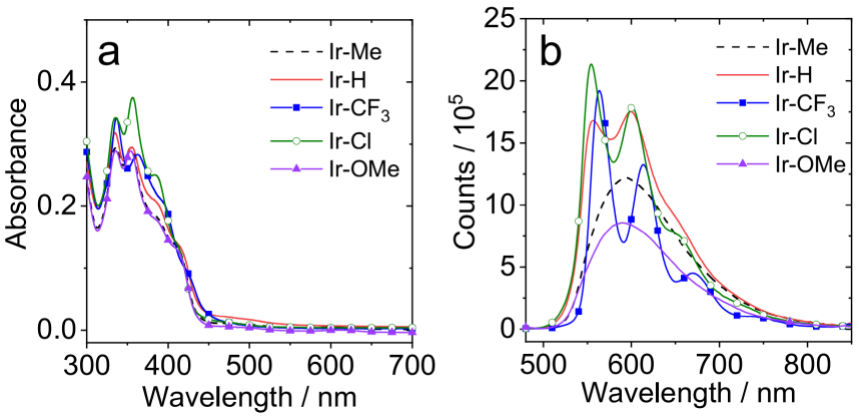
(a) UV/vis absorption spectra of the Ir(III) complexes in methanol, *c* = 1.0 × 10^–5^ M. (b) Phosphorescence emission spectra of the Ir(III) complexes in degassed dichloromethane (N_2_ atmosphere). Optically matched solutions were used in each panel (each of the solutions gives the same absorbance at the excitation wavelength, *A* = 0.100), λ_ex_ = 410 nm, 20 °C.

The UV–vis absorption data was obtained in aerated methanol and presented in Table 2 and Figure 2. The complexes absorb strongly (ε > 5000 M^–1^ cm^–1^) below 425 nm, which is primarily attributed to a combination of spin-allowed, ligand-centered transitions associated with the different aromatic constituents within the ligands. Quite pronounced vibronic features are superimposed upon the different absorption features, which is consistent with the highly planar naphthyl-based structures. A weaker shoulder feature at 400–450 nm is evident across the series of complexes and is attributed to a band comprising a metal-to-ligand charge transfer (MLCT) contribution. A much weaker (ε < 1000 M^–1^ cm^–1^) feature at 450–550 nm, which is most pronounced for **Ir–H**, is possibly due to a spin forbidden S_0_ → T_1_ transition mediated by the heavy Ir atom (which possesses a large spin–orbit coupling constant).^34^ Across the series of complexes, the absorption spectra are broadly comparable suggesting the type of substituent present in the cyclometalated ligand produces a very subtle effect in terms of the absorption character of the complexes. Placed in context, the absorption features of these complexes share some similarities with the ubiquitous [Ir(ppy)_2_(bipy)]^+^ (where ppy = 2-phenylpyridine),^35^ but with more efficient molar absorption in the 350–400 nm range, which is likely due to the more conjugated 2-phenyl-1*H*-naphtho[2,3-*d*]imidazole cyclometalating ligands.

**Table 2:** Absorption and Photoluminescence Data for the Complexes^a^

	σ_p_^36^	absorbance λ_max_/nm^c^ (ε/10^4^/M^–1^cm^–1^)^b^	emission λ/nm^c^	lifetime, τ_aerated_/μs(τ_deox_/μs)^d^	quantum yield, Φ/%^e^
**Ir–OMe**	–0.27	354 (2.9)	589	0.17, 0.27 (0.49, 1.2)	4.6
**Ir–Me**	–0.17	335 (2.9)	593	0.20, 0.29 (0.50, 1.1)	5.2
**Ir–H**	0	335 (3.1)	598	0.39, 0.40 (1.9, 3.3)	2.8
**Ir–Cl**	0.22	357 (3.8)	554	0.64 (10.0)	1.5
**Ir–CF**_**3**_	0.54	337 (3.4)	563	0.61 (7.4)	1.0

a All measurements obtained in methanol at 293 K.

b 1 × 10^4^ M^–1^cm^–1^.

c λ_ex_ = 410 nm.

d Observed photoluminescence lifetime, λ_ex_ = 403 nm.

e [Ru(bipy)_3_][PF_6_]_2_ serving as the reference in aerated MeCN, and the quantum yield (Φ) is 1.8%.

**Figure 3:**
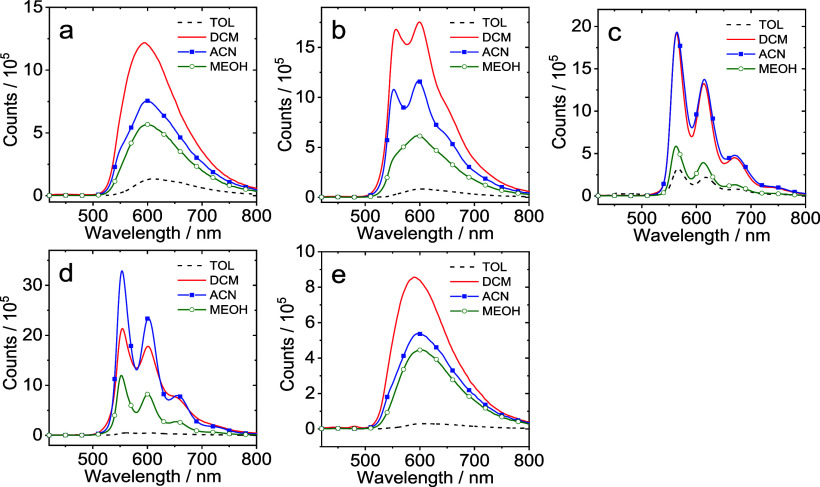
Phosphorescence emission spectra of the (a) **Ir–Me**, (b) **Ir–H**, (c) **Ir–CF**_**3**_, (d) **Ir–Cl**, and (e) **Ir–OMe** complexes in degassed solvents. Optically matched solutions were used in each panel (each of the solutions gives the same absorbance at the excitation wavelength, *A* = 0.100), λ_ex_ = 410 nm, 20 °C.

The photoluminescence properties of the complexes were assessed under a range of conditions (using excitation at 410 nm). Figure 3 shows a comparison of the spectra obtained from the complexes in degassed solutions (toluene, dichloromethane, acetonitrile, and methanol) at room temperature. Photoluminescence was found to be strongly quenched under aerated conditions (Figure S24). While the emission maxima for the complexes lie in the range of 554–598 nm, implying these are green-to-orange emitters (note that [Ir(ppy)_2_(bipy)]^+^ emits with a broad featureless peak at 602 nm assigned to an admixture of ^3^MLCT/^3^LLCT states), the appearance of the spectral profile varies profoundly across the series. Both **Ir–Me** and **Ir–OMe** display emission bands which are broad and featureless, whereas **Ir–Cl** and **Ir–CF**_**3**_ possess highly structured emission spectra. **Ir–H** was perhaps intermediate between the two, with somewhat dampened vibronic features which were also shown to be solvent sensitive.

Time-resolved luminescence studies (Figure S25) also revealed some interesting differences. First, **Ir–Cl** and **Ir–CF**_**3**_ possessed very similar aerated lifetimes of 604 and 638 ns which extend dramatically upon deoxygenation to ca. 7.4 and 10.0 μs, respectively. Together with the structured spectral appearance of the emission band, it appears that a ligand-centered triplet (^3^LC) emission most likely dominates for these two complexes. The decay profiles for aerated solutions of **Ir–Me** and **Ir–OMe** were apparently biexponential and typically <300 ns. Upon deoxygenation, the fitted decay profile again yielded two lifetime values which were both extended, but to a much lesser degree than **Ir–Cl** and **Ir–CF**_**3**_. **Ir–H** showed decay kinetics that lie between these two extremes. This data showed that the nature of the ligand substituent strongly influences the excited state character of the emitting state. In the case of **Ir–Cl** and **Ir–CF**_**3**_, the emission is thus assigned to a ^3^LC excited state localized on the cyclometalating ligand, while **Ir–Me** and **Ir–OMe** are probably dominated by^3^MLCT behavior, and **Ir–H** probably has a relatively increased admixture of ^3^LC character to the emitting state.

**Figure 4:**
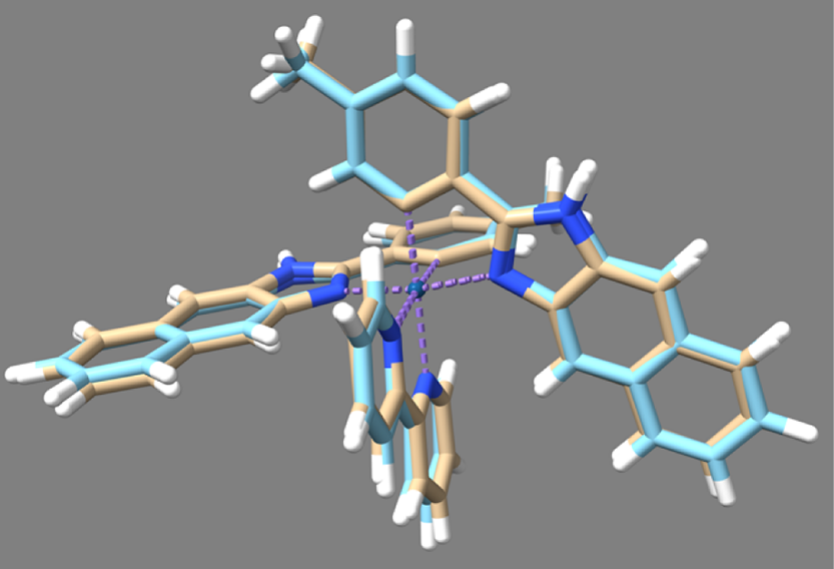
Overlay of the experimentally derived (beige) and calculated (DFT // B3LYP/6-31 G*) SDD optimized singlet (blue) structures of Ir-Me. RMSD = 0.253 Å.

Supporting computational studies (DFT) were deployed to further rationalize the experimental observations regarding the electronic properties of the complexes. All calculations were undertaken using the B3LYP functional with a 6-31G* basis set and an SDD ECP for the central iridium atom. The validity of the models were corroborated by comparing the minimum energy optimized geometries (Figure S26) with the crystallographic data; the DFT optimizations for **Ir–H**, **Ir–Me**, and **Ir–OMe** are in excellent agreement with the experimental crystal structures (Figure 4).

**Figure 5:**
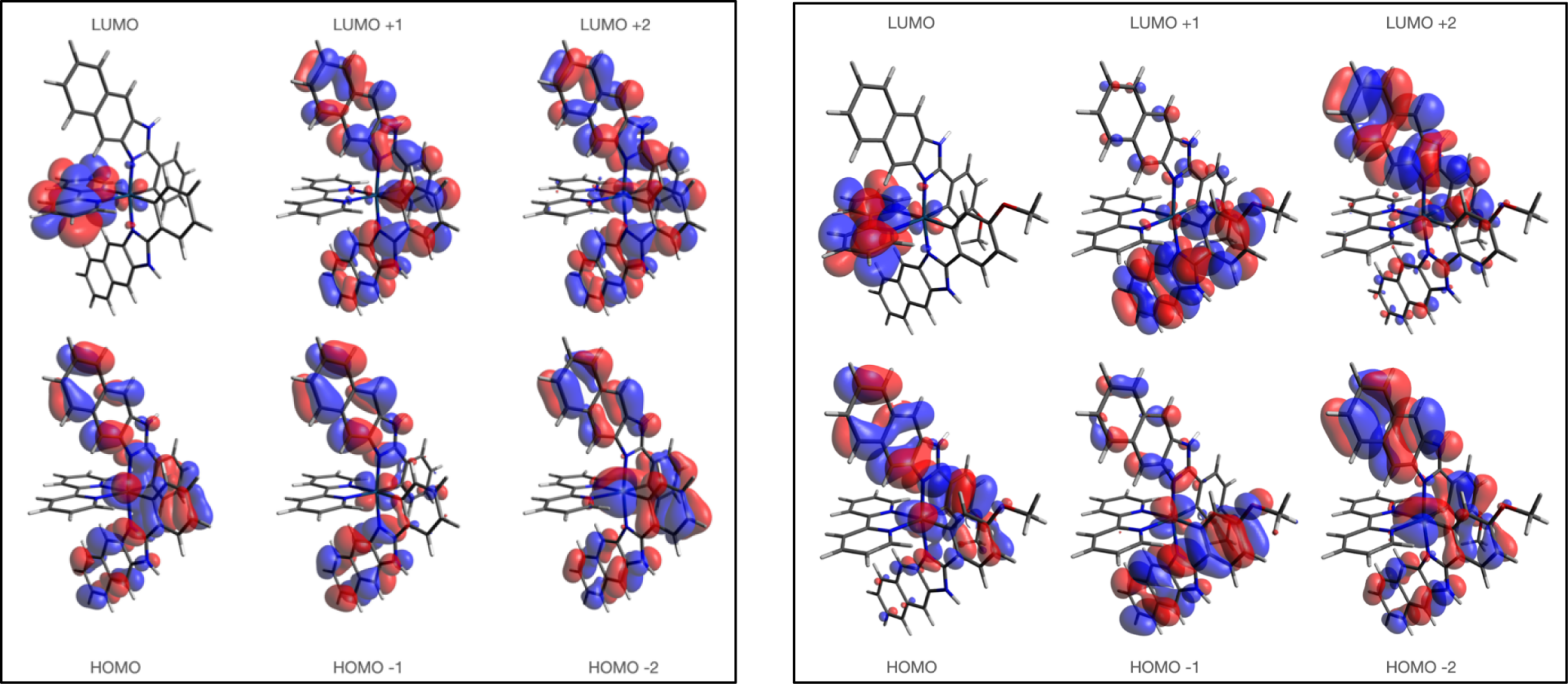
A comparison of the calculated Kohn–Sham frontier molecular orbitals for **Ir–H** (left) and **Ir–OMe** (right).

Molecular orbital decomposition analyses were performed in each case and predicted that the occupied orbitals have varying extents of Ir d-orbital and ligand-centered π character while the unoccupied orbitals are almost exclusively ligand-centered. Pictorial representations of the important frontier orbitals are shown in Figure 5 and S27–S29.

Although the calculations predict that the LUMO is located on the bipyridine ligand, it is not strongly represented in the transitions with the highest predicted oscillator strengths. In all cases, the calculations predict varying mixtures of MLCT (Ir → L_C^N_) and C^N ligand-centered orbital contributions to the excited state. The pattern of orbital contributions is very similar for **Ir–H** and **Ir–Me** where the key LUMO+1 is localized on the C^N ligands. Interestingly, for **Ir–OMe**, the calculations predict a loss of degeneracy across the two C^N ligands (Figure 5) and thus a mixture of both MLCT and LLCT may contribute to the excited state. DFT predicts that the introduction of an electron-withdrawing group (**Ir–Cl** and **Ir–CF**_**3**_) reduces the HOMO and HOMO–1 density at the metal, implying an overall weaker MLCT contribution, as noted in the experimental data. To estimate the vertical excitation energies of the low-lying singlet and triplet excited states of the complexes, TD-DFT calculations were carried out from the optimized ground-state geometries. The most important singlet transitions and their associated oscillator strengths are tabulated (Table 3 and Tables S3–S6) and suggest potential origins to the spectral features. The predicted spin forbidden S_0_ → T_1_ wavelengths also correlate well with the weak tail to the lowest energy absorption bands of the complexes. The calculations also performed quite well (Table 4) in the prediction of the T_1_ → S_0_ emission energies, with a clear correlation to the experimental data observed for both **Ir–Cl** and **Ir–CF**_**3**_ versus the other complexes in the series.

**Table 3:** Description of the Calculated MO Contributions, Excited States, and Their Associated Transitions for **Ir–H** Complex (L1 and L2 are the Cyclometalating Ligands; Bpy = 2,2′-bipyridine)

	moiety contribution (%)				orbital contribution to excited state	
orbital	Ir	Bpy	L1	L2	excited State	contributing transitions (>10%)
**LUMO +4**	2	96	1	1	1 (351.22 nm, *f* = 0.0051)	HOMO −2 → LUMO (31.18%)
**LUMO +3**	3	96	1	1		HOMO → LUMO (54.6%)
**LUMO +2**	2	2	48	48	2 (336.29 nm, *f* = 0.4174)	**HOMO −2 → LUMO +1 (25.8%)**
**LUMO +1**	1	0	49	49		HOMO −1 → LUMO +2 (12.3%)
						**HOMO → LUMO +1 (41.69%)**
**LUMO**	3	97	0	0	3 (334.14 nm, *f* = 0.0322)	HOMO −1 → LUMO (37.65%)
**HOMO**	21	1	39	39		HOMO −1 → LUMO +1 (10.85%)
						HOMO → LUMO +2 (22.3%)
**HOMO −1**	9	1	45	45	4 (327.66 nm, *f* = 0.187)	HOMO −1 → LUMO (42%)
**HOMO −2**	27	2	36	36		HOMO −1 → LUMO +1 (13.9%)
						HOMO → LUMO +2 (22.95%)
**HOMO −3**	3	1	48	48	5 (325.2 nm, *f* = 0.0031	HOMO −6 → LUMO (12.85%)
**HOMO −4**	18	1	41	41		HOMO −4 → LUMO (11.4%)
						HOMO −2 → LUMO (48.44%)
						HOMO → LUMO (22.88%)

**Table 4:** Computed Values for the Absorption and Emission Maxima of the Experimentally Isolated Ir(III) Complexes^a^

complex	S_0_ → S_1_/nm	S_0_ → T_1_/nm	T_1_ → S_0_/nm
**Ir–H**	336 (335)	465	569 (598)
**Ir–Me**	337 (335)	472	578 (593)
**Ir–OMe**	335 (354)	468	611 (589)
**Ir–Cl**	334 (357)	447	552 (554)
**Ir–CF**_**3**_	340 (337)	450	558 (563)

a Experimentally determined wavelength maxima shown in parentheses.

**Figure 6:**
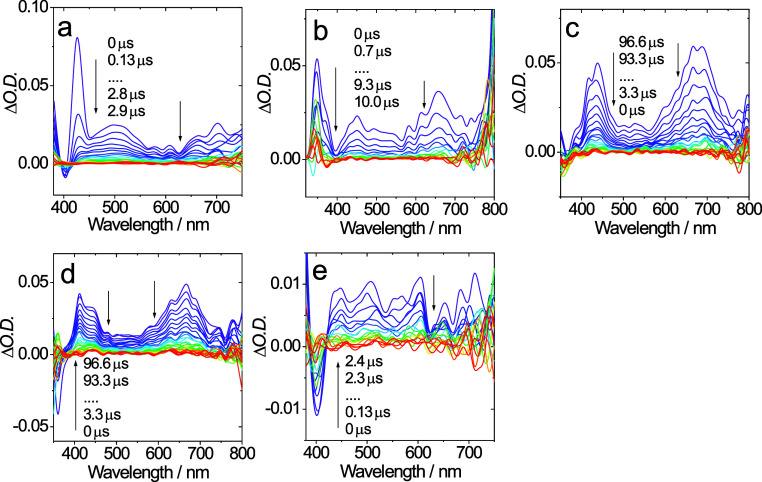
Nanosecond time-resolved transient absorption spectra for (a) **Ir–Me**, (b) **Ir–H**, (c) **Ir–CF**_**3**_, (d) **Ir–Cl**, and (e) **Ir–OMe** complexes in degassed dichloromethane under nitrogen atmosphere upon pulsed laser excitation, λ_ex_ = 420 nm, *c* = 3 × 10^–5^ M.

Transient absorption spectra (degassed CH_2_Cl_2_) were obtained (Figure 6) and corroborated the differences in the triplet character of the complexes. The data show the changes in optical density as a function of time following nanosecond pulsed laser irradiation at 420 nm. The spectra of **Ir–CF**_**3**_ and **Ir–Cl** showed two main positive excited state absorption features ca. 450 and 650 nm, whereas the other complexes look qualitatively different with several features across the 400–750 nm range. The decay kinetics (Figure S30) showed that both **Ir–CF**_**3**_ and **Ir–Cl** possess much longer time scale triplet characteristics compared to the other complexes, which aligns with the observations of the photoluminescent measurements and may imply that the transient absorption spectra arise from closely related excited states.^37^

**Figure 7:**
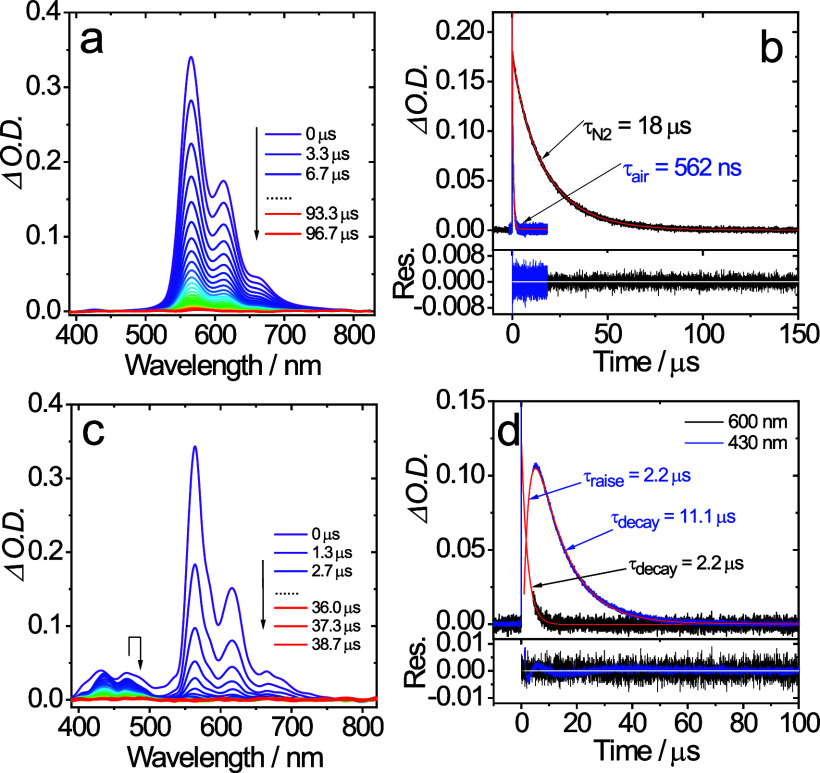
(a) Time-resolved luminescence of **Ir–CF**_**3**_ (*c* = 3.0 × 10^–5^ M); (b) the decay traces of phosphorescence in different atmosphere; (c) delayed fluorescence with **Ir–CF**_**3**_ (*c* = 3.0 × 10^–5^ M) as the triplet photosensitizer and DPA (*c* = 1.0 × 10^–4^ M) as the triplet acceptor; (d) the decay traces of the emission at 600 nm (T_1_ → S_0_) and 430 nm (^1^DPA* → S_0_); the spike in the delayed fluorescence traces is the scattered laser. Excited with nanosecond pulsed laser at 445 nm. In deaerated dichloromethane, 20 °C.

TTA-UC studies were undertaken assessing each of the Ir(III) complexes as a prospective photosensitizer and 9,10-diphenylanthracene (DPA) as the annihilator (the T_1_ level of DPA is ca. 700 nm and therefore lies below the triplet emitting level of all of the complexes herein). Time-resolved emission spectra were collected after sequential additions of DPA in deoxygenated CH_2_Cl_2_. In all cases, an excitation wavelength of 445 nm was used, which is selective for the visible absorption band of the complexes and avoids direct excitation of the DPA. Therefore, any emission from DPA in the 400–500 nm range can be solely attributed to an upconversion process via TTA resulting in delayed fluorescence. In summary, both **Ir–CF**_**3**_ and **Ir–Cl** were demonstrated to be viable photosensitizers for TTA-UC (Figures 7 and S31–S34). For example, the data for **Ir–CF**_**3**_ showed (Figure 9) that upon addition of DPA, the triplet emission at 550–750 nm was strongly quenched (and the lifetime reduced from 18.0 to 2.2 μs) and a new band was evident at 430 nm which is attributed to the delayed fluorescence of the DPA. The decay kinetics of this band showed two features: first, a grow-in phase which has a rise time of 2.2 μs, followed by a slow decay (τ = 11.1 μs). Thus, the rise time of the DPA emission corresponds to the accumulation of the DPA triplet state and the resultant decay of **Ir–CF**_**3**_ in the presence of DPA. The reduction in lifetime of triplet emission from **Ir–CF**_**3**_ is as expected if annihilation is assumed to be a diffusion-controlled process. In contrast, neither **Ir–Me** nor **Ir–OMe** was a viable photosensitizer for TTA-UC, suggesting that both complexes were clearly hindered by their comparatively short triplet lifetimes (Figures S31 and S32). In comparison, **Ir–H** produced weak, but definitive TTA-UC fluorescence, which we ascribe to the much shorter phosphorescence lifetime of the complex (2.3 μs, which is then quenched to <1 μs in the presence of DPA) (Figure S33). Finally, as predicted from the spectral data, **Ir–Cl** demonstrated broadly comparable behavior to **Ir–CF**_**3**_. The quenching of the photosensitizer triplet emission results in a substantive diminution of the lifetime from 24.0 to 2.7 μs (Figure S34); again, the rise time of the delayed fluorescence is comparable to the quenched lifetime of the photosensitizer.

From the time-resolved data, the rate constants for the triplet energy transfer were obtained directly from the fitted rise-times of the decay trace obtained at 430 nm (e.g., Figure 7d) giving *k*_ET_ = 4.5 × 10^5^ s^–1^, 5.3 × 10^5^ s^–1^, and 6.7 × 10^5^ s^–1^ for **Ir–CF**_**3**_, **Ir–Cl**, and **Ir–H**, respectively. Therefore, as **Ir–CF**_**3**_, **Ir–Cl**, and **Ir–H** each show a long triplet lifetime (>1.2 μs) this is beneficial to the triplet energy transfer to DPA and upconversion emission. Conversely, the relatively short triplet lifetimes of **Ir–Me** and **Ir–OMe** render triplet energy transfer less favorable because ^3^MLCT→ S_0_ competes very effectively.

## Conclusions

This paper describes the synthesis and characterization of a series of luminescent iridium(III) complexes that incorporate cyclometalated imidazole ligands prepared from 2,3-diaminonaphthalene. The point of structural variance is a substituent on the metalated phenyl ring, providing either electron-donating or withdrawing capacity to this donor moiety. Three of the complexes were fully characterized in the solid state using single crystal X-ray diffraction. Both steady state and time-resolved photophysical studies unanimously revealed the phosphorescent nature of the complexes, but with significant variation noted within the series, which in turn depended upon the type of ligand substituent. This approach allowed rational and exquisite tuning between either a predominant ^3^MLCT or ^3^LC character to the emitting state of these complexes (which was further supported by DFT). The series of complexes was then assessed as photosensitizers in TTA-UC using DPA as the annihilator species. Our results showed that the Ir(III) complexes with the longest triplet lifetimes (those with a dominant ^3^LC character) performed best as photosensitizers for TTA-UC, therefore suggesting that the use of electron-withdrawing substituents on the phenyl group in this ligand type is a convenient strategy for optimizing photosensitizer performance in TTA-UC.

## Supporting Information Available

HRMS, NMR, and IR spectra for all compounds; data collection parameters for the X-ray crystal structures; cyclic voltammetry data and additional DFT and photophysical data (PDF)
