# Supplementary material for: Tuning Excited State Character in Iridium(III) Photosensitizers and Its Influence on TTA-UC
Source: Inorg Chem. 2024 May 13;63(21):9931–40. doi: 10.1021/acs.inorgchem.4c01003 (PMC11134496; doi:10.1021/acs.inorgchem.4c01003)
Supplement: Supplementary file 1 — ic4c01003_si_001.pdf [file ic4c01003_si_001.pdf]

Supplementary Information

Tuning Excited State Character in Iridium(III) Photosensitizers

And its Influence on TTA-UC

Ibrahim S. Alkhaibari,[a,b] Xue Zhang,[c] Jianzhang Zhao,[c] Thomas M. Stonelake,[a] Richard C. Knighton,[d] Peter N. Horton,[e] Simon J. Coles,[e] Niklaas J. Buurma,[a] Emma Richards,[a] and Simon J. A. Pope[a]*

[a]School of Chemistry, Main Building, Cardiff University, Cardiff, CF10 3AT, Cymru/Wales, UK; [b] Department of Chemistry, College of Science, Qassim University, Buraydah 52571, Saudi Arabia; [c] State Key Laboratory of Fine Chemicals, Frontiers Science Center for Smart Materials, School of Chemical Engineering, Dalian University of Technology, Dalian 116024, P.R. China; [d] School of Chemistry, University of Southampton, Highfield, Southampton, SO17 1BJ, UK; [e]UK National Crystallographic Service, Chemistry, Faculty of Natural and Environmental Sciences, University of Southampton, Highfield, Southampton, SO17 1BJ, UK.

Email: popesj@cardiff.ac.uk

	Experimental procedures for the ligands (**L1-L5**) and characterization data	4-5
Figure S1	^{1}H NMR spectrum of 2-phenyl-1H-naphtho[2,3-d]imidazole (**L1**)	6
Figure S2	^{13}C NMR spectrum of 2-phenyl-1H-naphtho[2,3-d]imidazole (**L1**)	6
Figure S3	^{1}H NMR spectrum of 2-(p-tolyl)-1H-naphtho[2,3-d]imidazole (**L2**)	7
Figure S4	^{13}C NMR spectrum of 2-(p-tolyl)-1H-naphtho[2,3-d]imidazole (**L2**)	7
Figure S5	^{1}H NMR spectrum of 2-(4-methoxyphenyl)-1H-naphtho[2,3-d]imidazole (**L3**)	8
Figure S6	^{13}C NMR spectrum of 2-(4-methoxyphenyl)-1H-naphtho[2,3-d]imidazole (**L3**)	8
Figure S7	^{1}H NMR spectrum of 2-(4-chlorophenyl)-1H-naphtho[2,3-d]imidazole (**L4**)	9
Figure S8	^{13}C NMR spectrum of 2-(4-chlorophenyl)-1H-naphtho[2,3-d]imidazole (**L4**)	9
Figure S9	^{1}H NMR spectrum of 2-(4-(trifluoromethyl)phenyl)-1H-naphtho[2,3-d]imidazole (**L5**)	10
Figure S10	Left: ^{13}C NMR spectrum of 2-(4-(trifluoromethyl)phenyl)-1H-naphtho[2,3-d]imidazole (**L5**); right, the ^{19}F NMR spectrum.	10
Figure S11	^{1}H NMR spectrum of [Ir(2-phenyl-1H-naphtho[2,3-d]imidazole)$_2$(2,2'-bipyridine)][PF$_6$] (**Ir-H**)	11
Figure S12	^{13}C NMR spectrum of [Ir(2-phenyl-1H-naphtho[2,3-d]imidazole)$_2$(2,2'-bipyridine)][PF$_6$] (**Ir-H**)	12
Figure S13	^{1}H NMR spectrum of [Ir(2-(p-tolyl)-1H-naphtho[2,3-d]imidazole)$_2$(2,2'-bipyridine)][PF$_6$] (**Ir-Me**)	13

Figure S14	^{13}C NMR spectrum of [Ir(2-(p-tolyl)-1H-naphtho[2,3-d]imidazole)$_2$(2,2'-bipyridine)][PF_6] (**Ir-Me**)	14
Figure S15	^{1}H NMR spectrum of [Ir(2-(4-methoxyphenyl)-1H-naphtho[2,3-d]imidazole)$_2$(2,2'-bipyridine)][PF_6] (**Ir-OMe**)	15
Figure S16	^{13}C NMR spectrum of [Ir(2-(4-methoxyphenyl)-1H-naphtho[2,3-d]imidazole)$_2$(2,2'-bipyridine)][PF_6] (**Ir-OMe**)	16
Figure S17	^{1}H NMR spectrum of [Ir(2-(2-(4-chlorophenyl)-1H-naphtho[2,3-d]imidazole)$_2$(2,2'-bipyridine)][PF_6] (**Ir-Cl**)	17
Figure S18	^{13}C NMR spectrum of [Ir(2-(2-(4-chlorophenyl)-1H-naphtho[2,3-d]imidazole)$_2$(2,2'-bipyridine)][PF_6] (**Ir-Cl**)	18
Figure S19	^{1}H NMR spectrum of [Ir(2-(4-(trifluoromethyl)phenyl)-1H-naphtho[2,3-d]imidazole)$_2$(2,2'-bipyridine)][PF_6] (**Ir-CF$_3$**)	19
Figure S20	^{13}C NMR spectrum of [Ir(2-(4-(trifluoromethyl)phenyl)-1H-naphtho[2,3-d]imidazole)$_2$(2,2'-bipyridine)][PF_6] (**Ir-CF$_3$**)	20
Figure S21	^{19}F NMR spectrum of [Ir(2-(4-(trifluoromethyl)phenyl)-1H-naphtho[2,3-d]imidazole)$_2$(2,2'-bipyridine)][PF_6] (**Ir-CF$_3$**)	20
Figure S22	HR MS data for the five Ir(III) complexes.	21
Figure S23	Cyclic voltammetry of the complexes	24
Figure S24	Photoluminescence spectra of the (a) **Ir-Me**, (b) **Ir-H**, (c) **Ir-CF$_3$**, (d) **Ir-Cl** and (e) **Ir-OMe** complexes in dichloromethane under different atmospheres	25
Figure S25	Decay curves of photoluminescence for (a) **Ir-Me**, (b) **Ir-H**, (c) **Ir-CF$_3$**, (d) **Ir-Cl** and (e) **Ir-OMe** complexes in dichloromethane under different atmospheres	26
Figure S26	Overlay of the calculated singlet (beige) and triplet (blue) geometries of the complexes.	27
Figure S27	A comparison of the calculated Kohn−Sham frontier molecular orbitals for **Ir-Me**.	28
Figure S28	A comparison of the calculated Kohn−Sham frontier molecular orbitals for **Ir-Cl**.	28
Figure S29	A comparison of the calculated Kohn−Sham frontier molecular orbitals for **Ir-CF$_3$**.	29
Figure S30	TAS triplet state lifetime for (a) **Ir-Me**, (b) **Ir-H**, (c) **Ir-CF$_3$**, (d) **Ir-Cl** and (e) **Ir-OMe** complexes in dichloromethane under different atmospheres	31
Figure S31	(a) Time-resolved luminescence of **Ir-Me**. (b) No delayed fluorescence with **Ir-Me** as the triplet photosensitizer and DPA as the triplet acceptor. (c) The decay traces of the emission at 600 nm.	32
Figure S32	(a) Time-resolved luminescence of **Ir-OMe**. (b) No delayed fluorescence with **Ir-OMe** as the triplet photosensitizer and DPA as the triplet acceptor. (c) The decay traces of the emission at 600 nm	32
Figure S33	(a) Time-resolved luminescence of **Ir-H**. (b) The decay traces of phosphorescence in different atmosphere. (c) Delayed fluorescence with **Ir-H**	33

	as the triplet photosensitizer and DPA as the triplet acceptor. (d) The decay traces of the emission at 600 nm ($T_1 \rightarrow S_0$) and 430 nm (^{1}**DPA*** $\rightarrow S_0$)	
Figure S34	(a) Time-resolved luminescence of **Ir-Cl**. (b) The decay traces of phosphorescence in different atmosphere. (c) Delayed fluorescence with **Ir-Cl** as the triplet photosensitizer and DPA as the triplet acceptor. (d) The decay traces of the emission at 600 nm ($T_1 \rightarrow S_0$) and 430 nm (^{1}**DPA*** $\rightarrow S_0$)	34
Table S1	The data collection parameters from the X-ray crystallography	22
Table S2	Selected bond angles	23
Table S3	Description of the calculated MO contributions, excited states and their associated transitions for **Ir-Me** (L1 and L2 are the cyclometalating ligands; Bpy = 2,2'-bipyridine)	29
Table S4	Description of the calculated MO contributions, excited states and their associated transitions for **Ir-OMe** (L1 and L2 are the cyclometalating ligands; Bpy = 2,2'-bipyridine)	30
Table S5	Description of the calculated MO contributions, excited states and their associated transitions for **Ir-Cl** (L1 and L2 are the cyclometalating ligands; Bpy = 2,2'-bipyridine)	30
Table S6	Description of the calculated MO contributions, excited states and their associated transitions for **Ir-CF$_3$** (L1 and L2 are the cyclometalating ligands; Bpy = 2,2'-bipyridine)	31

General procedure for imidazole-naphthalene ligands

In a 100 mL round-bottom flask, 3.0 mmol of the aldehyde derivative was added to 25 mL of absolute ethanol, along with 0.7 mmol of ammonium chloride or 0.3 mmol of sodium metabisulfate. This mixture was stirred at room temperature for 15 minutes. Following that, 3.5 mmol of 2,3-diaminonaphthalene in 15 mL of absolute ethanol was added to the reaction mixture and then heated to reflux.[1] After 12 hours, the reaction mixture was placed into approximately 30 mL of water and stirred for a total of 20 minutes. After this, the desired compound was filtered and washed several times with water. Recrystallization from dichloromethane and methanol (70:30) was used to purify the crude solid.

2-phenyl-1H-naphtho[2,3-d]imidazole (L1) [2]

Isolated as a beige powder; (639 mg, 75%) ^{1}H NMR (500 MHz, d6-DMSO) δ: 12.97 (br, 1H, NH), 8.32 (d, J_{HH} = 8.3 Hz, 2H), 8.13 (s, 2H), 8.02 (q, J_{HH} = 8.0 Hz, 2H), 7.63-7.55 (m, 3H,), 7.38 (app. q, J_{HH} = 7.3 Hz, 2H); ^{13}C{^{1}H} NMR (126 MHz, d6-DMSO) δ: 155.8, 131.1, 130.5, 130.2, 129.5, 128.2, 127.5, 123.9 ppm; LR MS (ES+): *m*/*z* calcd 244.10 for $C_{17}H_{12}N_2$; found 245.11 $[M + H]^+$. IR (ATR, cm^{-1}): 3053 (NH), 1636 (C=N), 1543 (C=C), 1465 (C-N), 1417 (C-C).

2-(p-tolyl)-1H-naphtho[2,3-d]imidazole (L2) [3]

Isolated as a beige powder; (688 mg, 77%) ^{1}H NMR (500 MHz, d6-DMSO) δ: 12.88 (s, 1H, NH), 8.20 (d, J_{HH} = 9.6 Hz, 3H), 8.00 (s, 3H), 7.40 (d, J_{HH} = 8.4 Hz, 2H), 7.37- 7.34 (br, 2H), 2.40 (s, 3H, CH_3) ppm; ^{13}C{^{1}H} NMR (126 MHz, d6-DMSO) δ: 156.0, 144.9, 141.0, 136.3, 130.7-130.2 (overlapping), 130.0, 128.5-127.8 (overlapping), 127.5, 124.1-123.4, 115.4, 106.8, 21.5 (CH_3) ppm; LR MS (GC): *m*/*z* calcd 258.12 for $C_{18}H_{14}N_2$; found 258.11. IR (ATR, cm^{-1}): 3043 (NH), 2912 (C-H), 1612 (C=N), 1550 (C=C), 1492 (C-N), 1421 (C-C).

2-(4-methoxyphenyl)-1H-naphtho[2,3-d]imidazole (L3)[4]

Isolated as a dark beige powder; (770 mg, 82%) ^{1}H NMR (400 MHz, d6-DMSO) δ: 12.80 (s, 1H, NH), 8.24 (d, J_{HH} = 8.2 Hz, 2H), 8.15 (br, 1H), 8.00- 7.95 (s, 3H), 35.5 (s, 2H), 7.16 (d, J_{HH} = 7.1 Hz, 2H), 3.86 (s, 3H, CH_3) ppm; ^{13}C{^{1}H} NMR (101 MHz, d6-DMSO) δ: 161.2, 155.4, 144.5, 135.9, 130.1-129.7, 128.7, 127.9-127.3, 123.5-122.9,

122.2, 114.6-114.4, 106.1, 55.4 (CH_3) ppm; LR MS (ES+): *m*/*z* calcd 274.11 for $C_{18}H_{14}N_2O$; found 275.12 $[M + H]^+$. IR (ATR, cm^{-1}): 3007 (NH), 1637 (C=N), 1610 (C=C), 1462 (C-N), 1423 (C-C), 1246 (C-O).

2-(4-chlorophenyl)-1H-naphtho[2,3-d]imidazole (L4)[4]

Beige powder; (760 mg, 79%) ^{1}H NMR (300 MHz, d6-DMSO) δ: 13.03 (s, 1H, NH), 8.29 (d, J_{HH} = 8.2 Hz, 2H), 8.15 (br, 1H), 8.01 (q, J_{HH} = 8.0 Hz, 3H), 7.68 (d, J_{HH} = 7.6 Hz, 2H), 7.38 (q, J_{HH} = 7.3 Hz, 2H) ppm; ^{13}C{^{1}H} NMR (126 MHz, d6-DMSO) δ: 154.2, 144.2, 135.7, 135.3 (C-Cl), 130.4-129.8, 129.1-128.8, 128.6, 128.1-127.4, 123.8-123.1, 115.3, 106.6 ppm; LR MS (ES+): *m*/*z* calcd 278.06 for $C_{17}H_{11}ClN_2$; found 279.07 $[M + H]^+$. IR (ATR, cm^{-1}): 3067 (NH), 1602 (C=N), 1583 (C=C), 1473 (C-N), 1446 (C-C), 738 (C-Cl).

2-(4-(trifluoromethyl)phenyl)-1H-naphtho[2,3-d]imidazole (L5)

Isolated as a light beige powder; (980 mg, 93%) ^{1}H NMR (500 MHz, d6-DMSO) δ: 13.20 (s, 1H, NH), 8.50 (d, J_{HH} = 8.1 Hz, 2H), 8.27 (s, 1H), 8.05- 8.01(m, 3H), 7.97 (d, J_{HH} = 8.5 Hz, 2H), 7.42- 7.35 (m, 2H) ppm; ^{13}C{^{1}H} NMR (126 MHz, d6-DMSO) δ: 153.7, 144.1, 135.7, 133.6-130.3 (q, $^2J_{C-F}$ = 32.7 Hz), 129.9-128.1, 127.7, 127.4, 126.0 (q, $^3J_{C-F}$ = 4.0 Hz), 124.0 (q, $^1J_{C-F}$ = 273.4 Hz), 123.2-123.0, 115.8, 106.9 ppm; ^{19}F{^{1}H} NMR (471 MHz, d6-DMSO) δ: -61.29 (s, CF_3) ppm; HR MS (ES+): *m*/*z* calcd 312.09 for $C_{18}H_{11}F_3N_2$; found 313.0955 $[M + H]^+$. IR (ATR, cm^{-1}): 3415 (NH), 1620 (C=N), 1541 (C=C), 1446 (C-N), 1425 (C-C), 1112 (C-F).

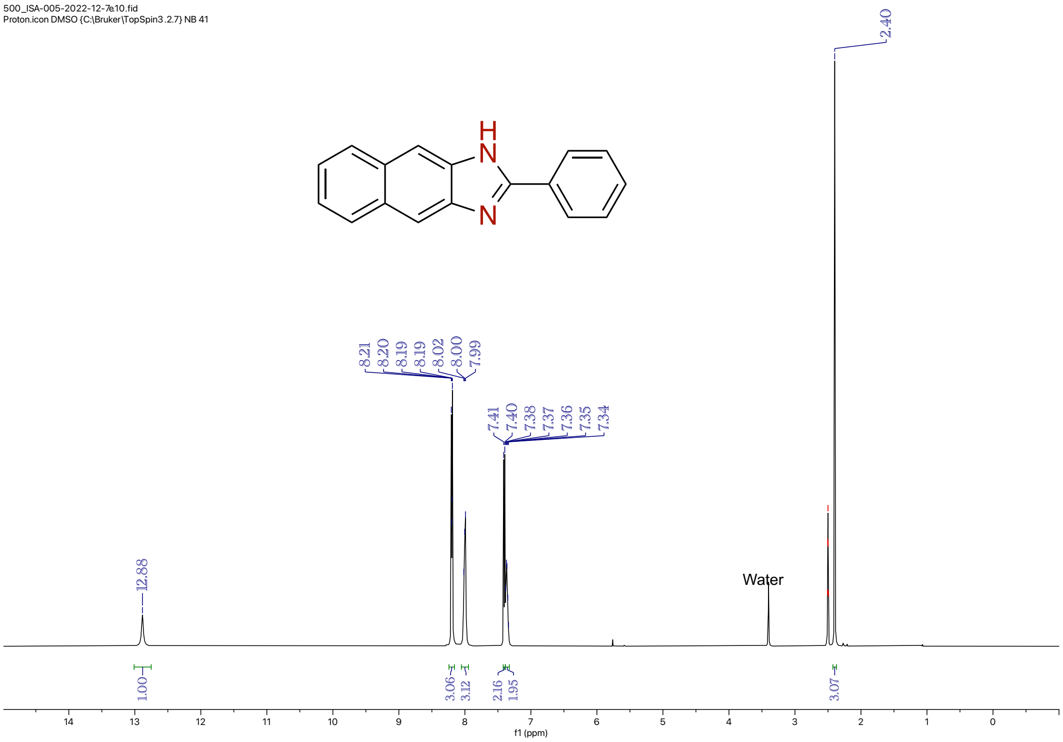

Figure S1. ^{1}H NMR spectrum of 2-phenyl-1H-naphtho[2,3-d]imidazole (L1)

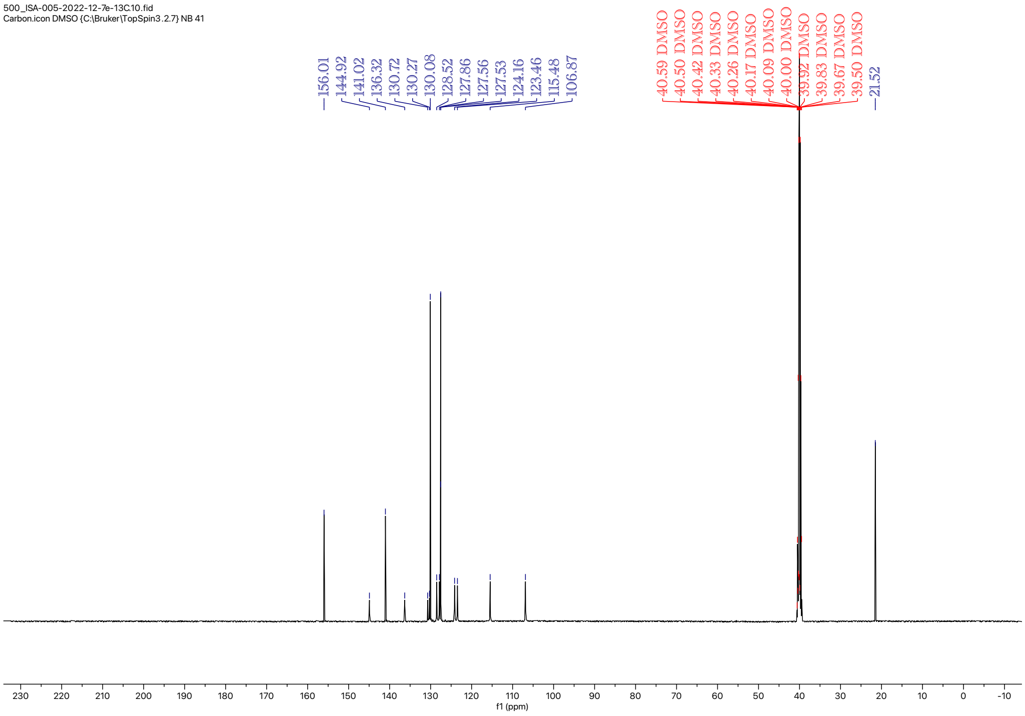

Figure S2. $^{13}C\{^{1}H\}$ NMR spectrum of 2-phenyl-1H-naphtho[2,3-d]imidazole (L1)

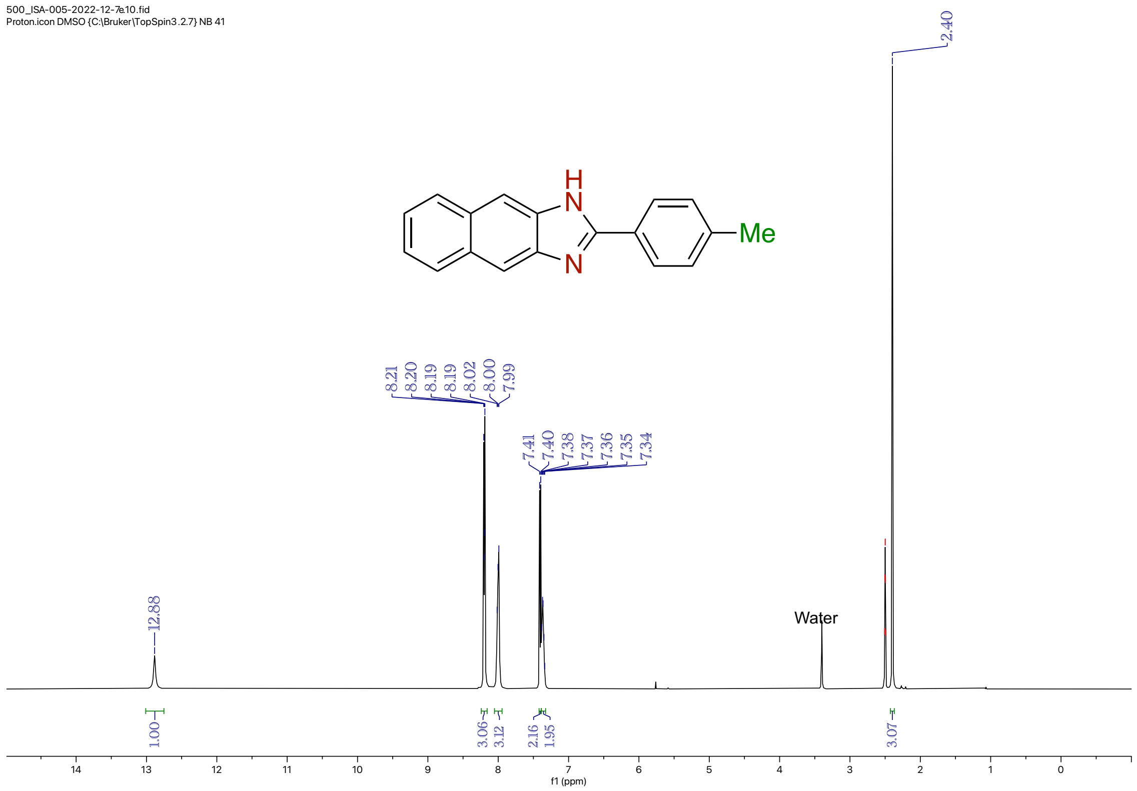

Figure S3. ^{1}H NMR spectrum of 2-(p-tolyl)-1H-naphtho[2,3-d]imidazole (L2)

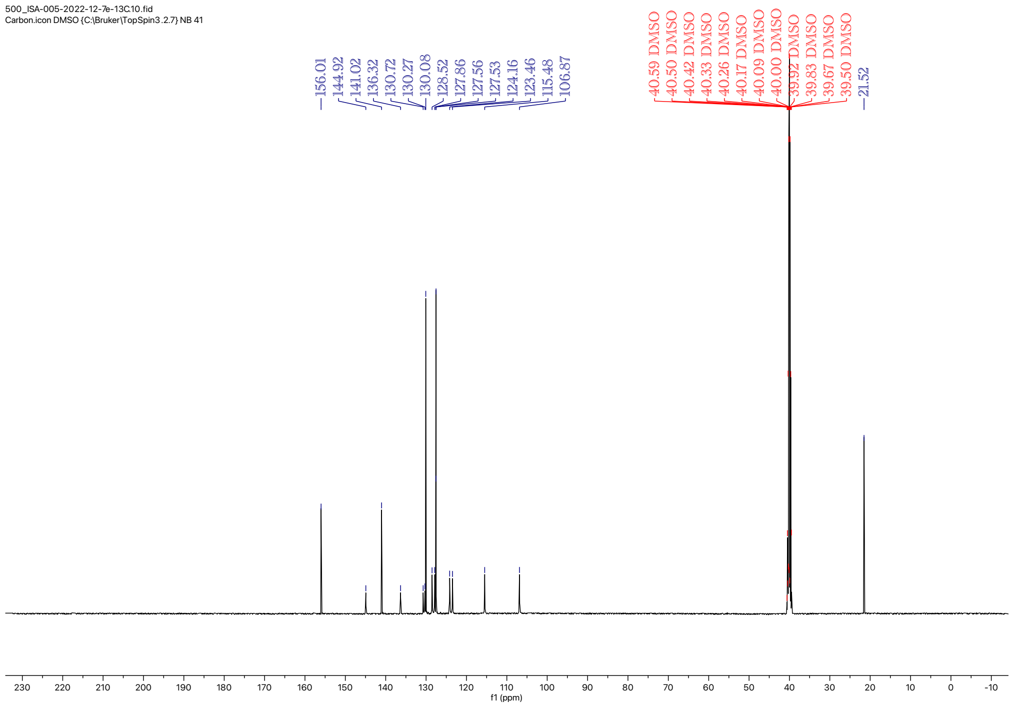

Figure S4. ^{13}C{^{1}H} NMR spectrum of 2-(p-tolyl)-1H-naphtho[2,3-d]imidazole (L2)

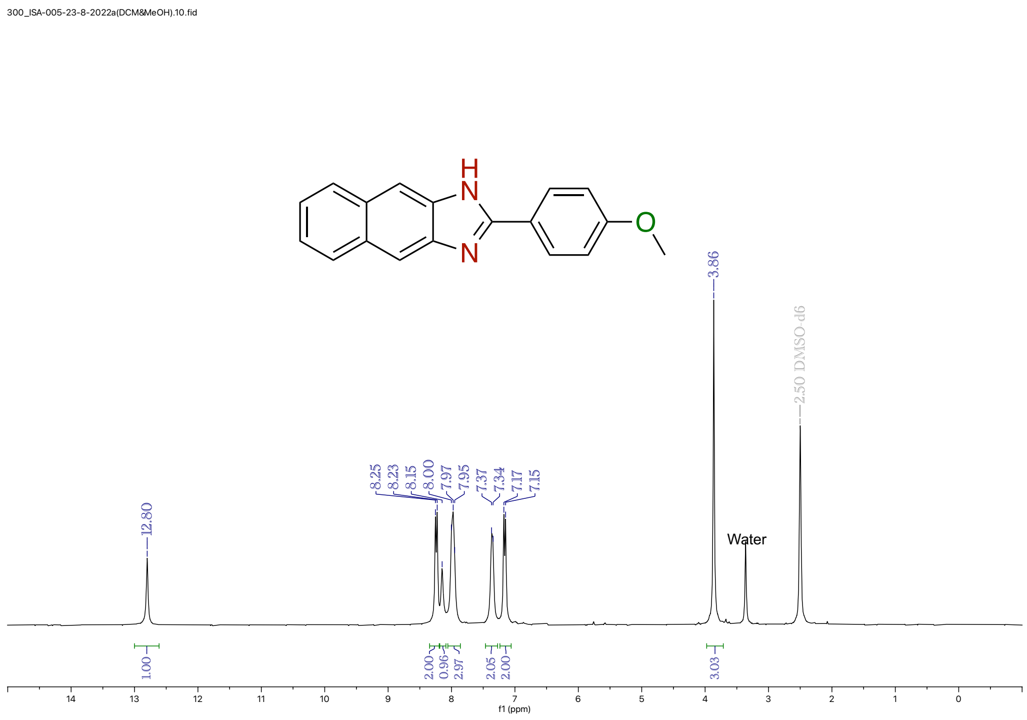

Figure S5. ^{1}H NMR spectrum of 2-(4-methoxyphenyl)-1H-naphtho[2,3-d]imidazole (L3)

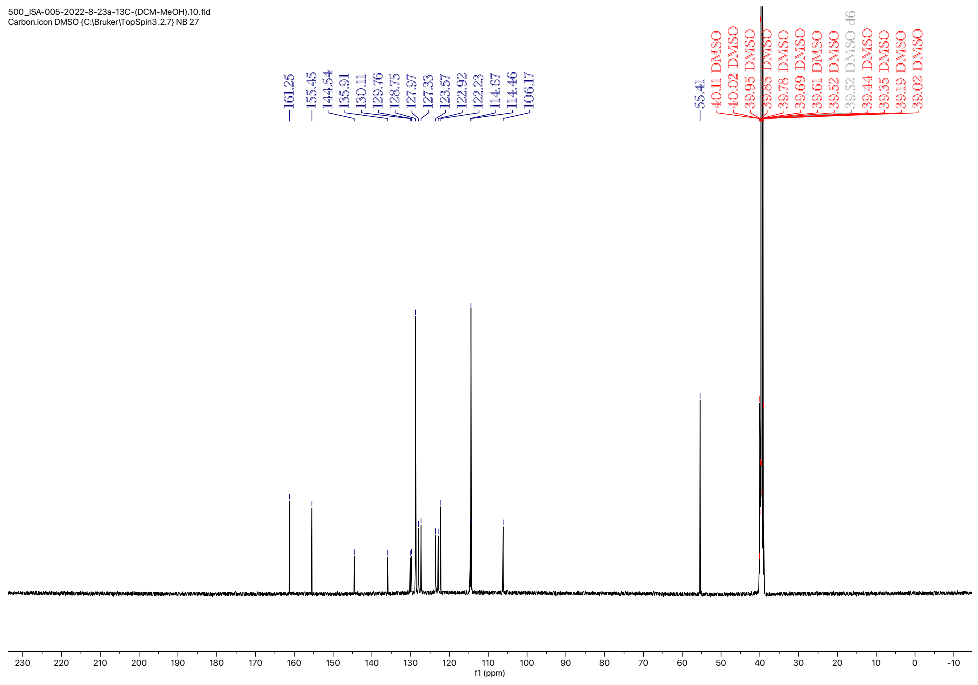

Figure S6. $^{13}C\{^{1}H\}$ NMR spectrum of 2-(4-methoxyphenyl)-1H-naphtho[2,3-d]imidazole (L3)

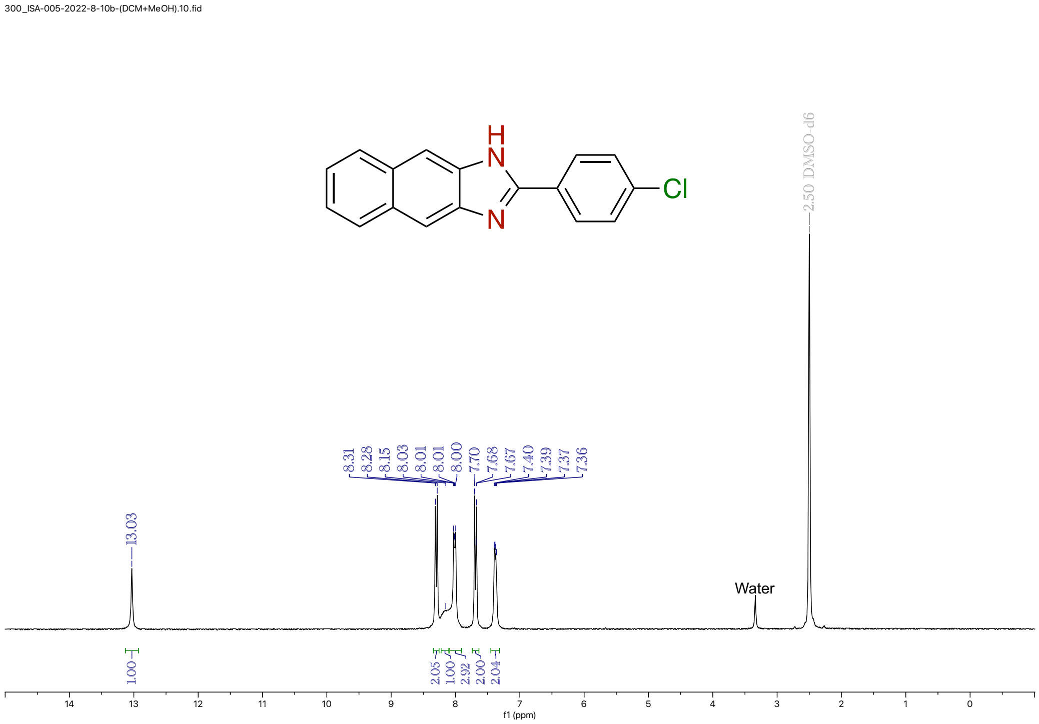

Figure S7. ^{1}H NMR spectrum of 2-(4-chlorophenyl)-1H-naphtho[2,3-d]imidazole (L4)

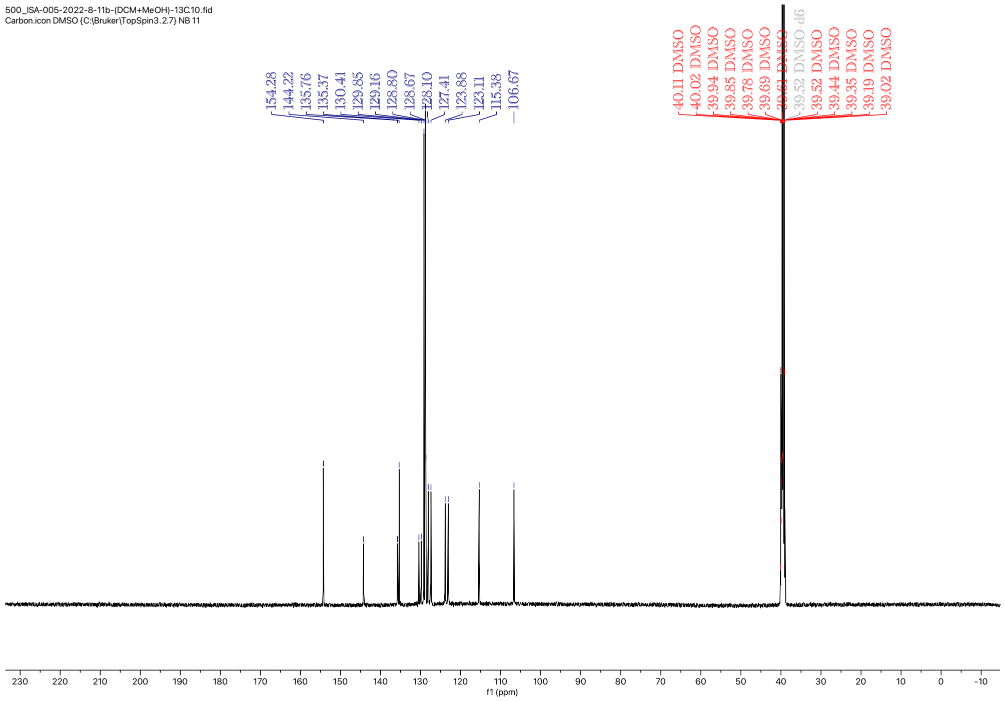

Figure S8. $^{13}C\{^1H\}$ NMR spectrum of 2-(4-chlorophenyl)-1H-naphtho[2,3-d]imidazole (L4)

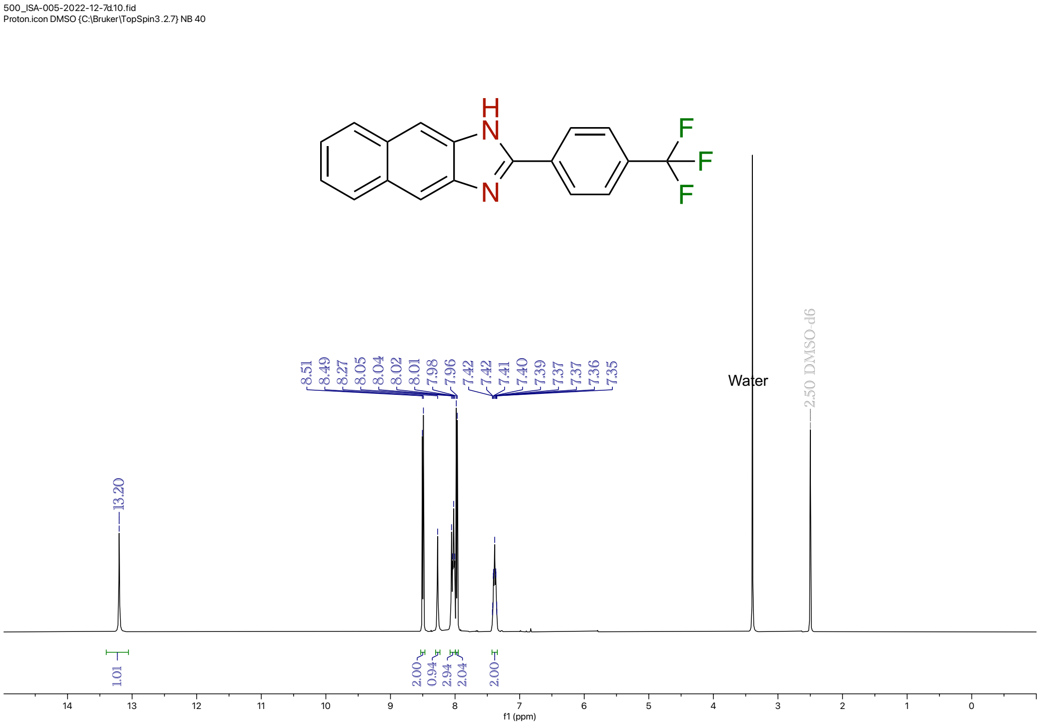

Figure S9. ^{1}H NMR spectrum of 2-(4-(trifluoromethyl)phenyl)-1H-naphtho[2,3-d]imidazole (L5)

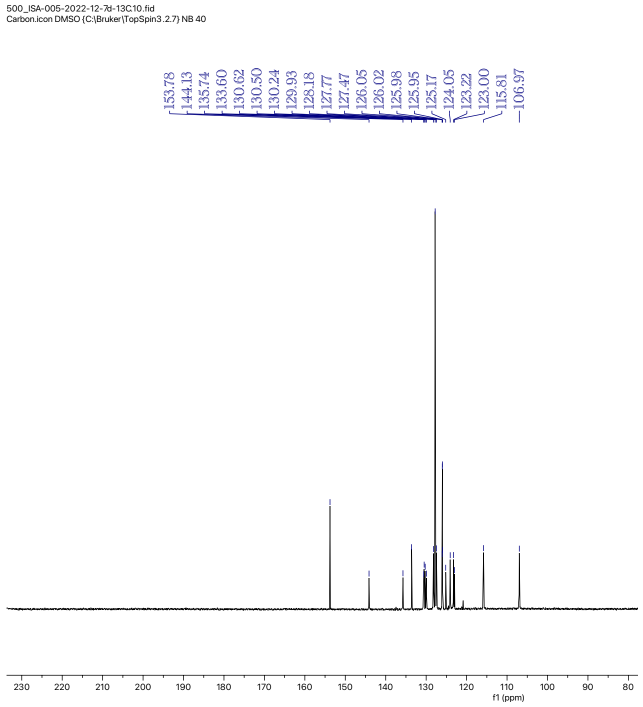

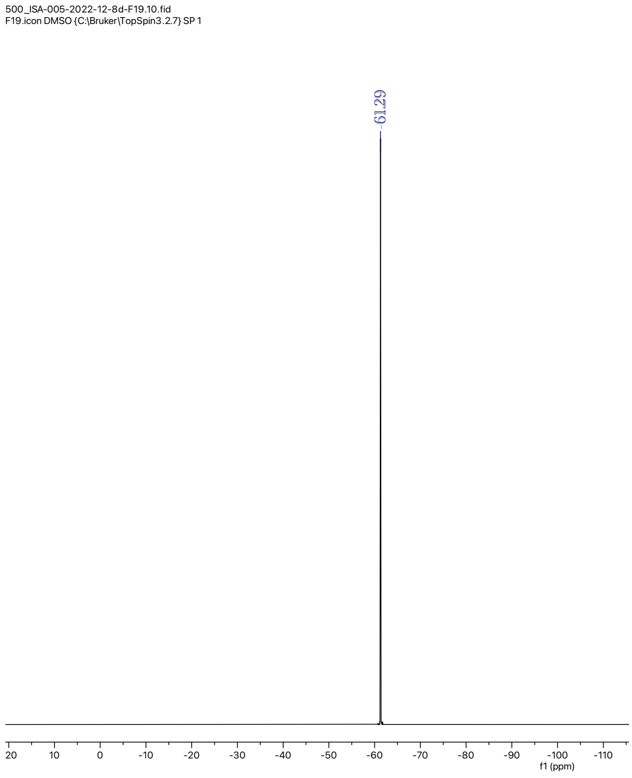

Figure S10. Left: $^{13}C\{^1H\}$ NMR spectrum of 2-(4-(trifluoromethyl)phenyl)-1H-naphtho[2,3-d]imidazole (L5); right, the $^{19}F\{^1H\}$ NMR spectrum.

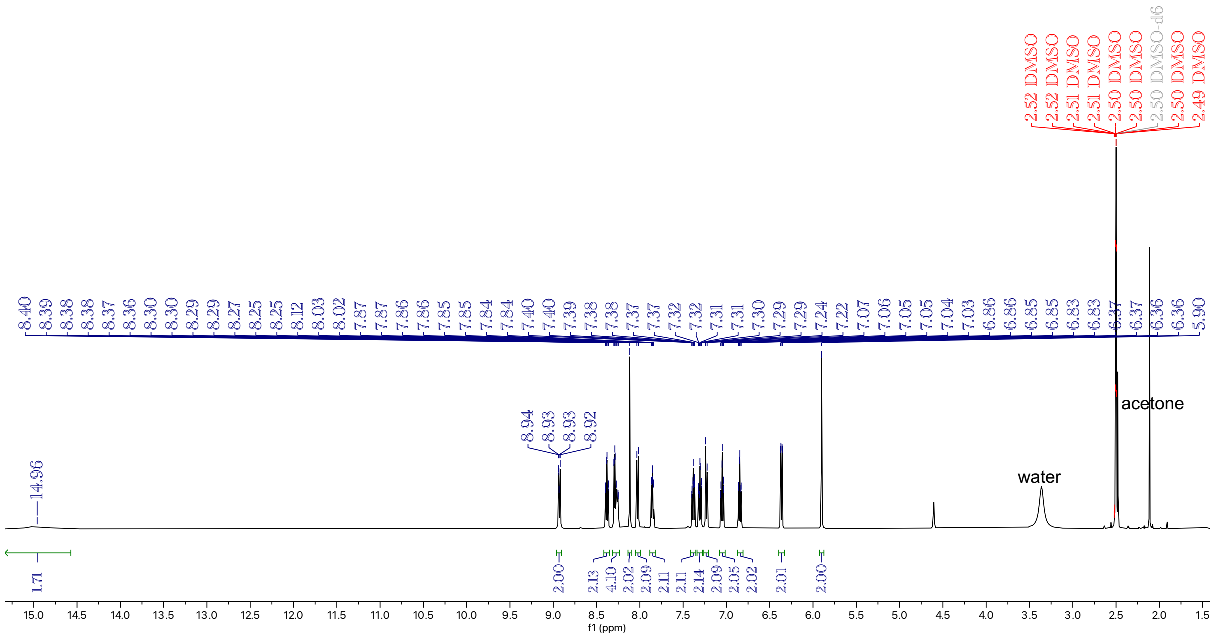

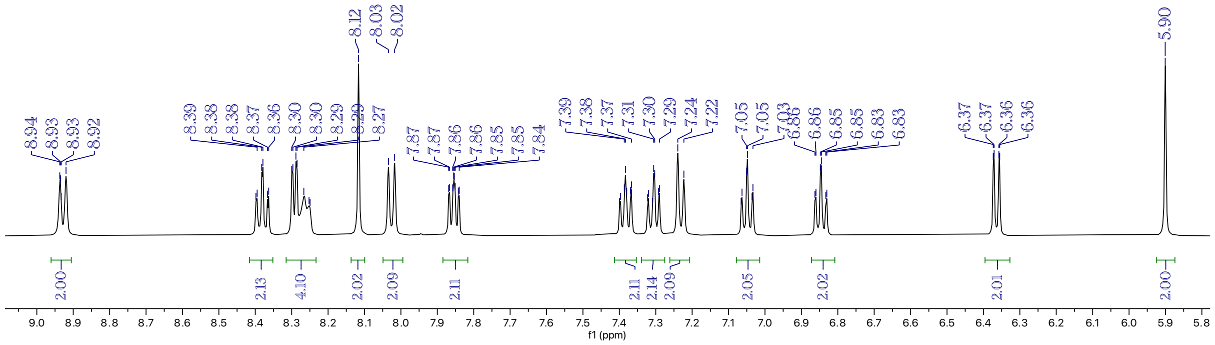

Figure S11. ^{1}H NMR spectrum of [Ir(2-phenyl-1H-naphtho[2,3-d]imidazole)$_2$(2,2'-bipyridine)][PF_6] (Ir-H)

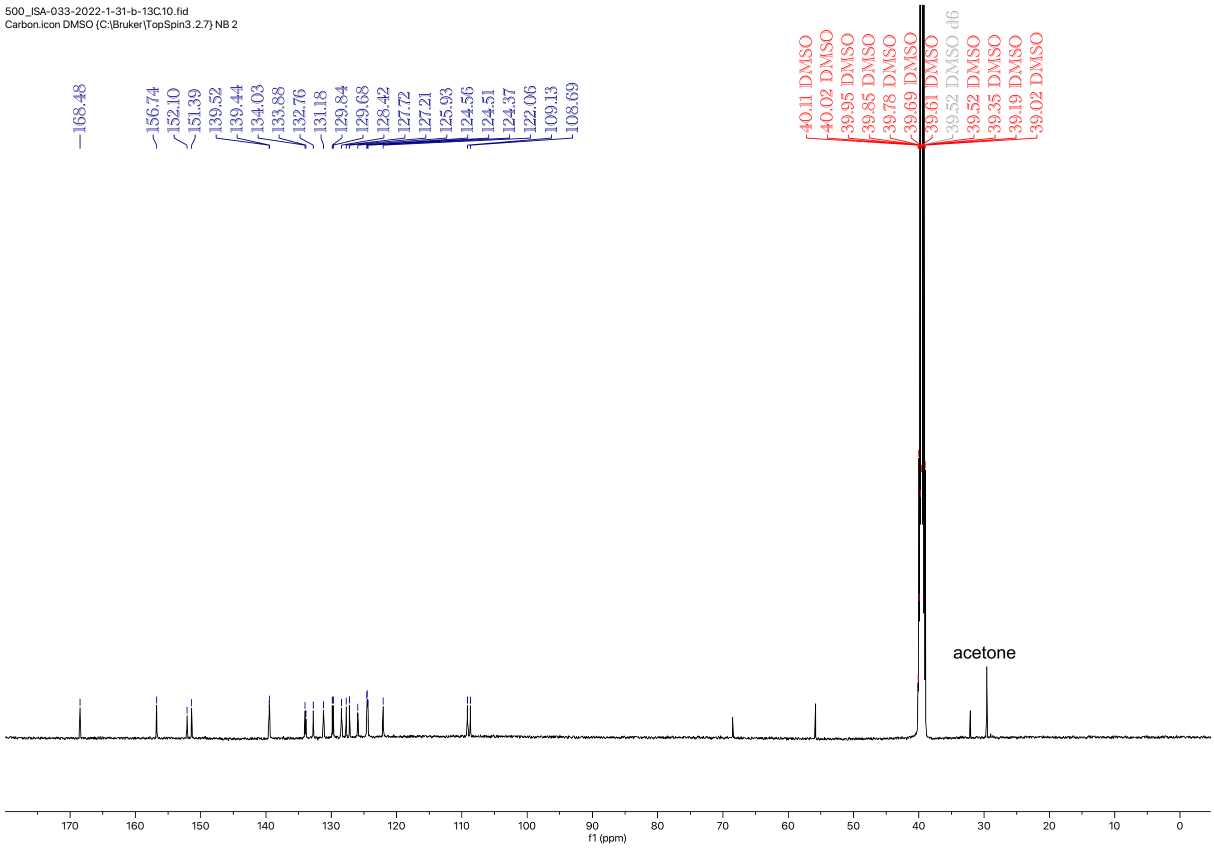

Figure S12. $^{13}C\{^{1}H\}$ NMR spectrum of [Ir(2-phenyl-1H-naphtho[2,3-d]imidazole)$_2$(2,2'-bipyridine)][PF_6] (Ir-H)

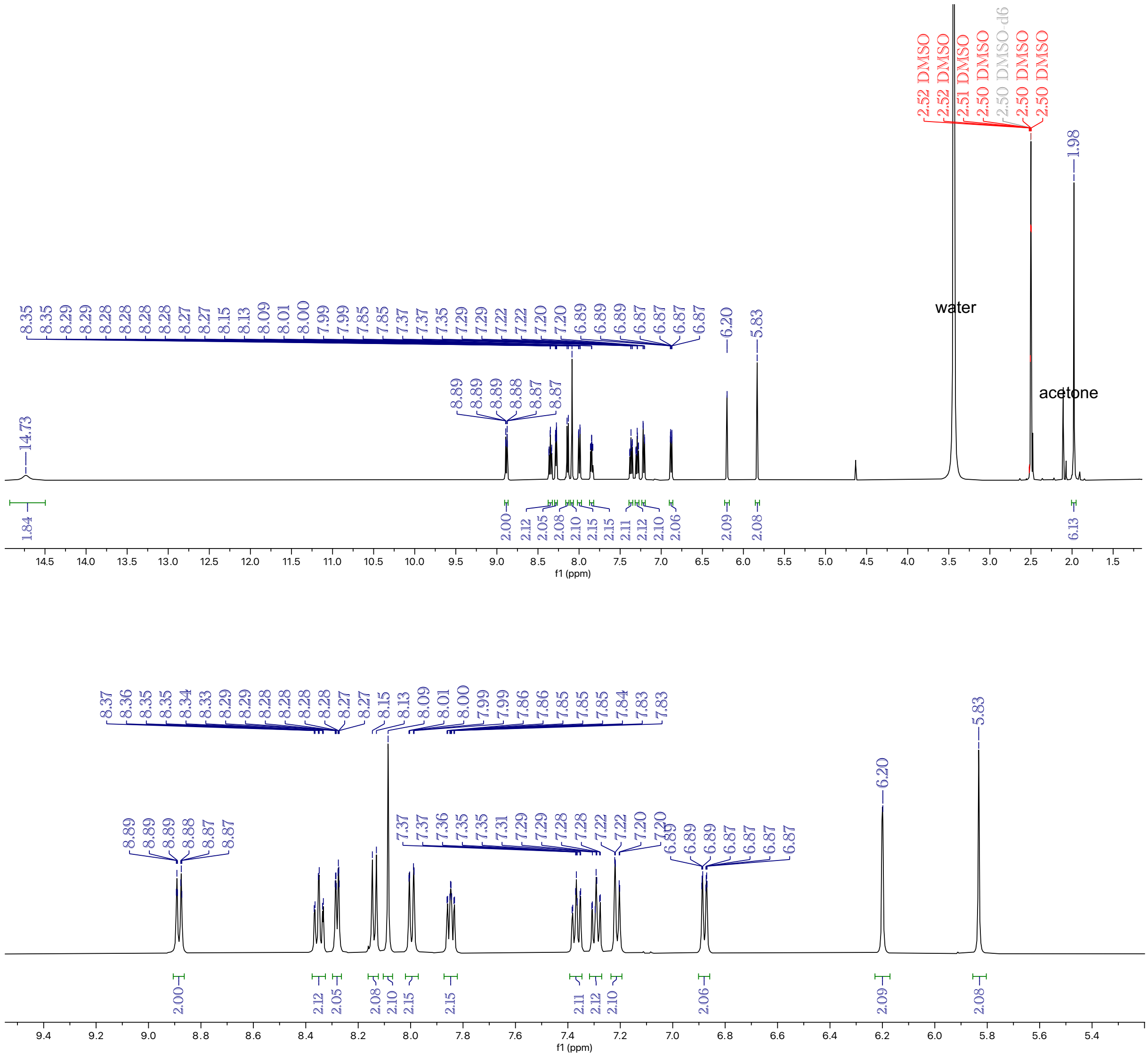

Figure S13. ^{1}H NMR spectrum of [Ir(2-(p-tolyl)-1H-naphtho[2,3-d]imidazole)$_2$(2,2'-bipyridine)][PF_6] (Ir-Me)

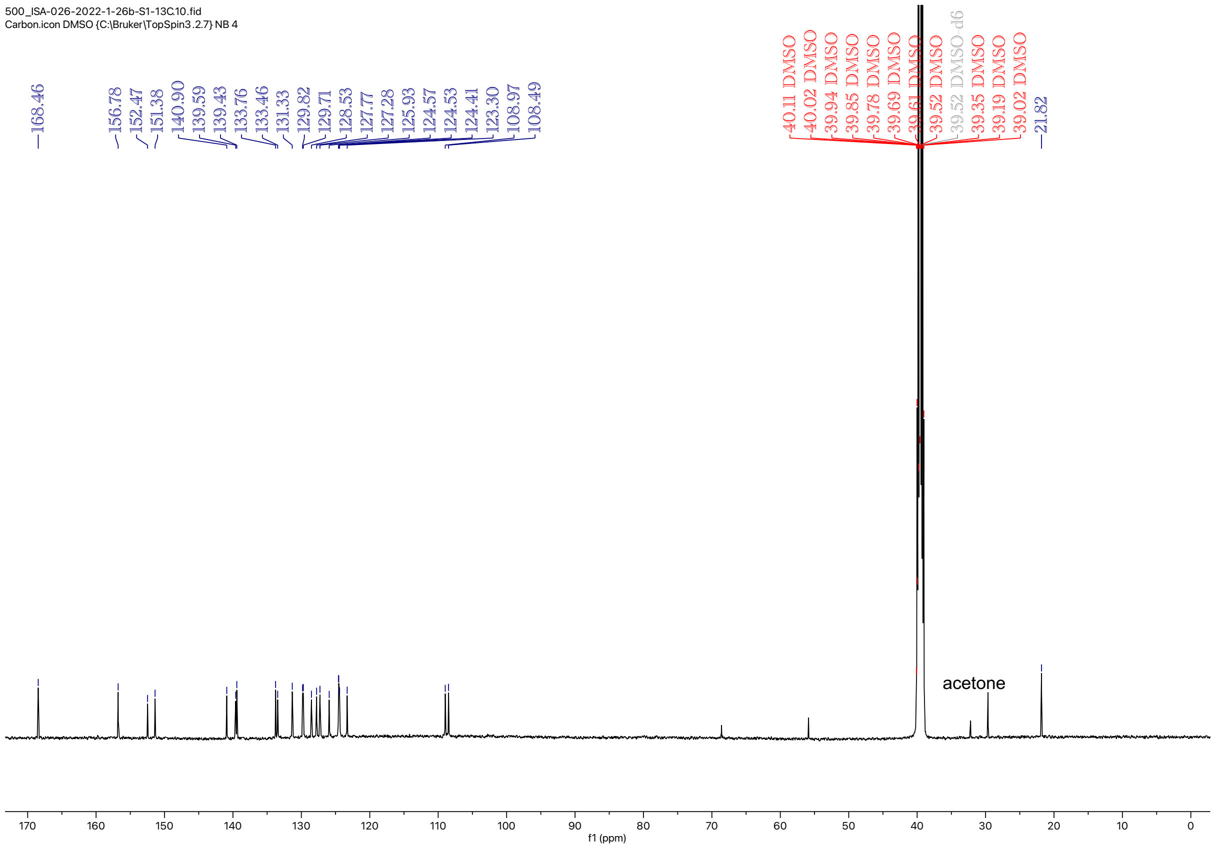

Figure S14. $^{13}C\{^{1}H\}$ NMR spectrum of [Ir(2-(p-tolyl)-1H-naphtho[2,3-d]imidazole)$_2$(2,2'-bipyridine)][PF_6] (Ir-Me)

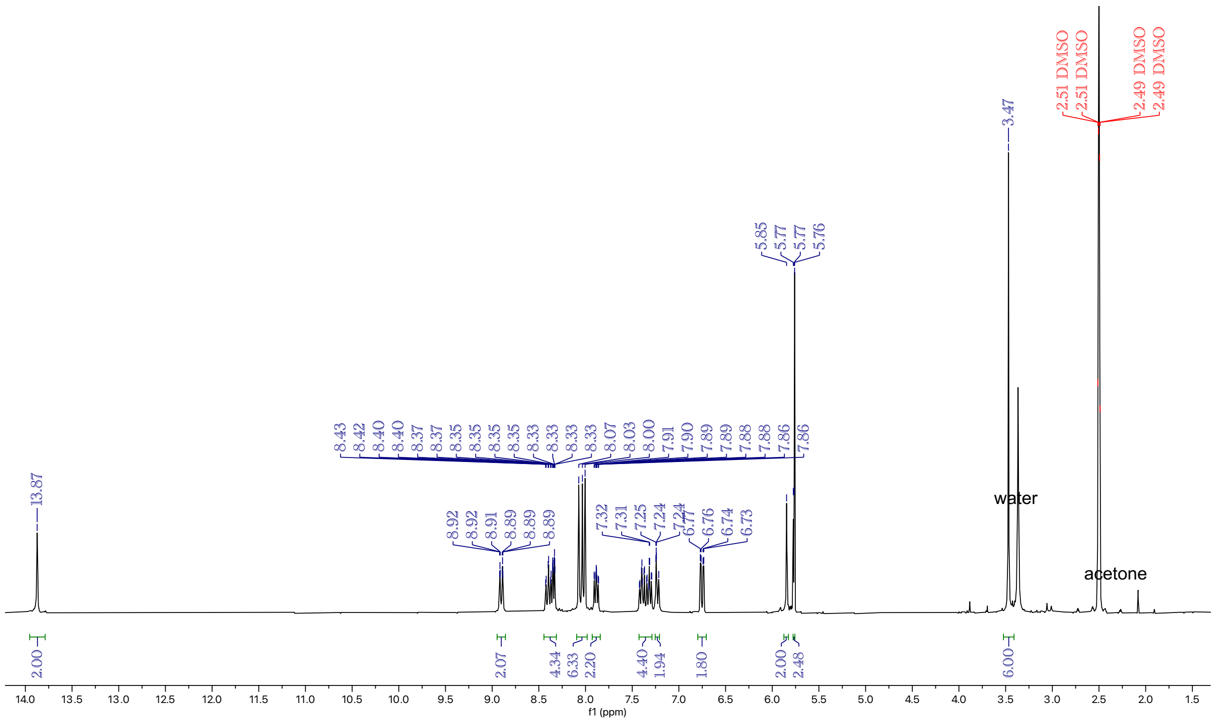

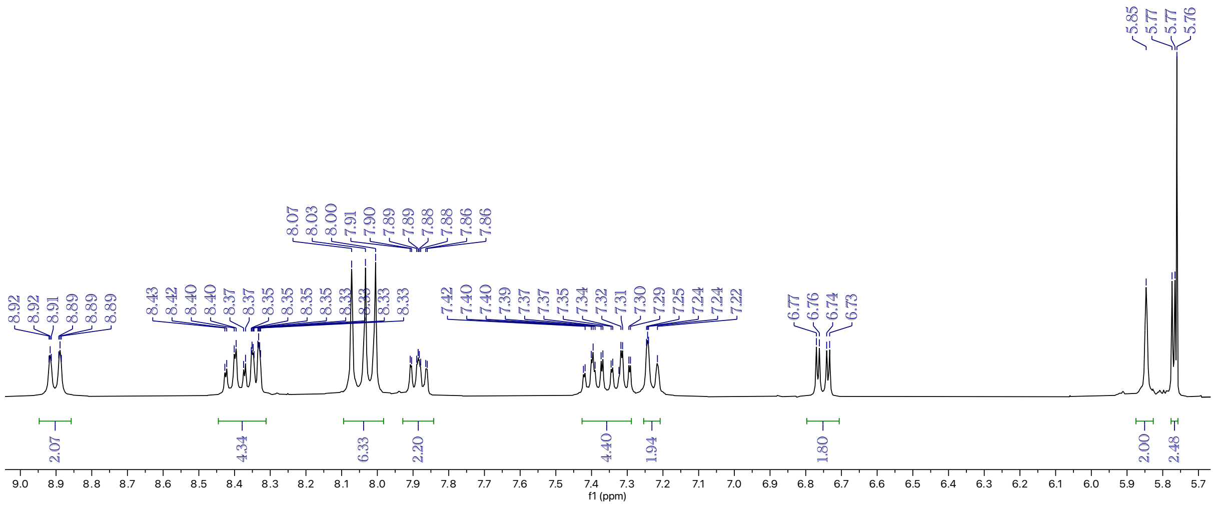

Figure S15. ^{1}H NMR spectrum of [Ir(2-(4-methoxyphenyl)-1H-naphtho[2,3-d]imidazole)$_2$(2,2'-bipyridine)][PF_6] (Ir-OMe)

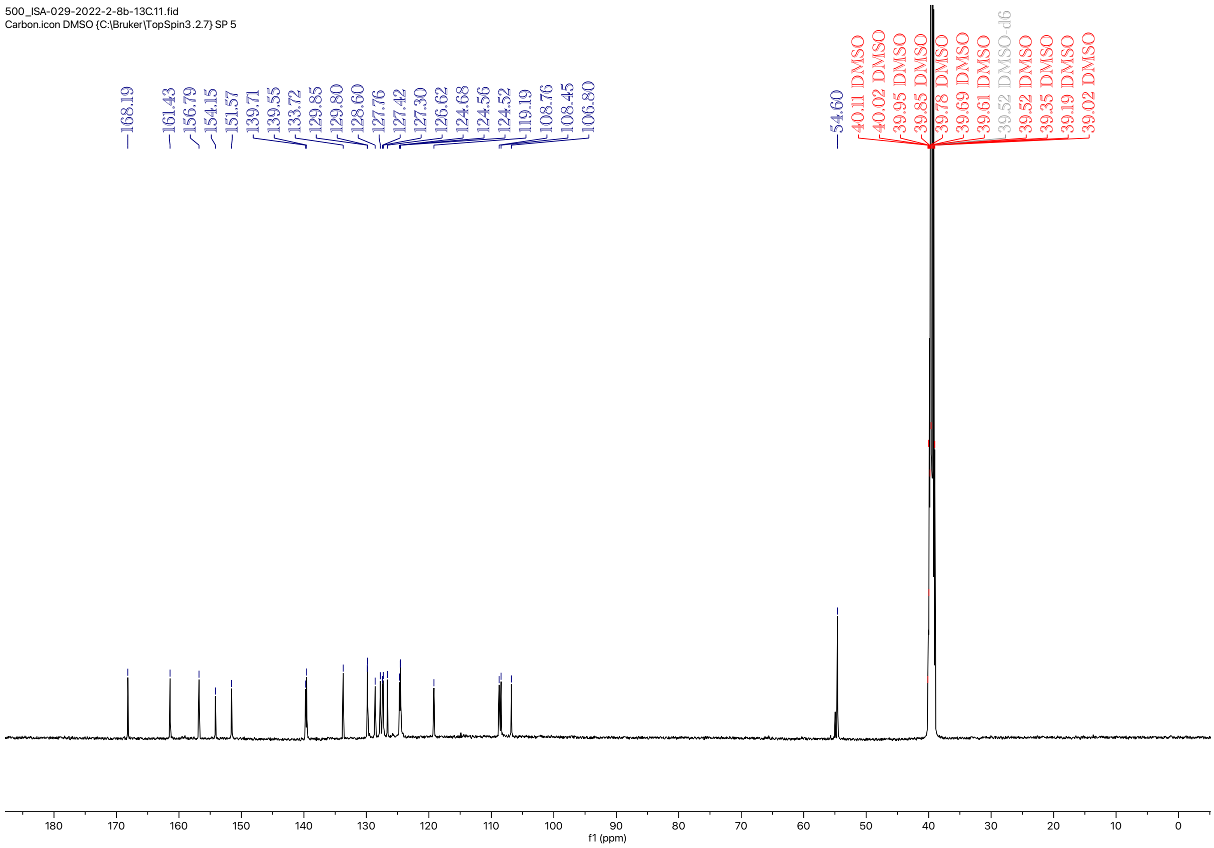

Figure S16. $^{13}C\{^1H\}$ NMR spectrum of [Ir(2-(4-methoxyphenyl)-1H-naphtho[2,3-d]imidazole)$_2$(2,2'-bipyridine)][PF_6] (Ir-OMe)

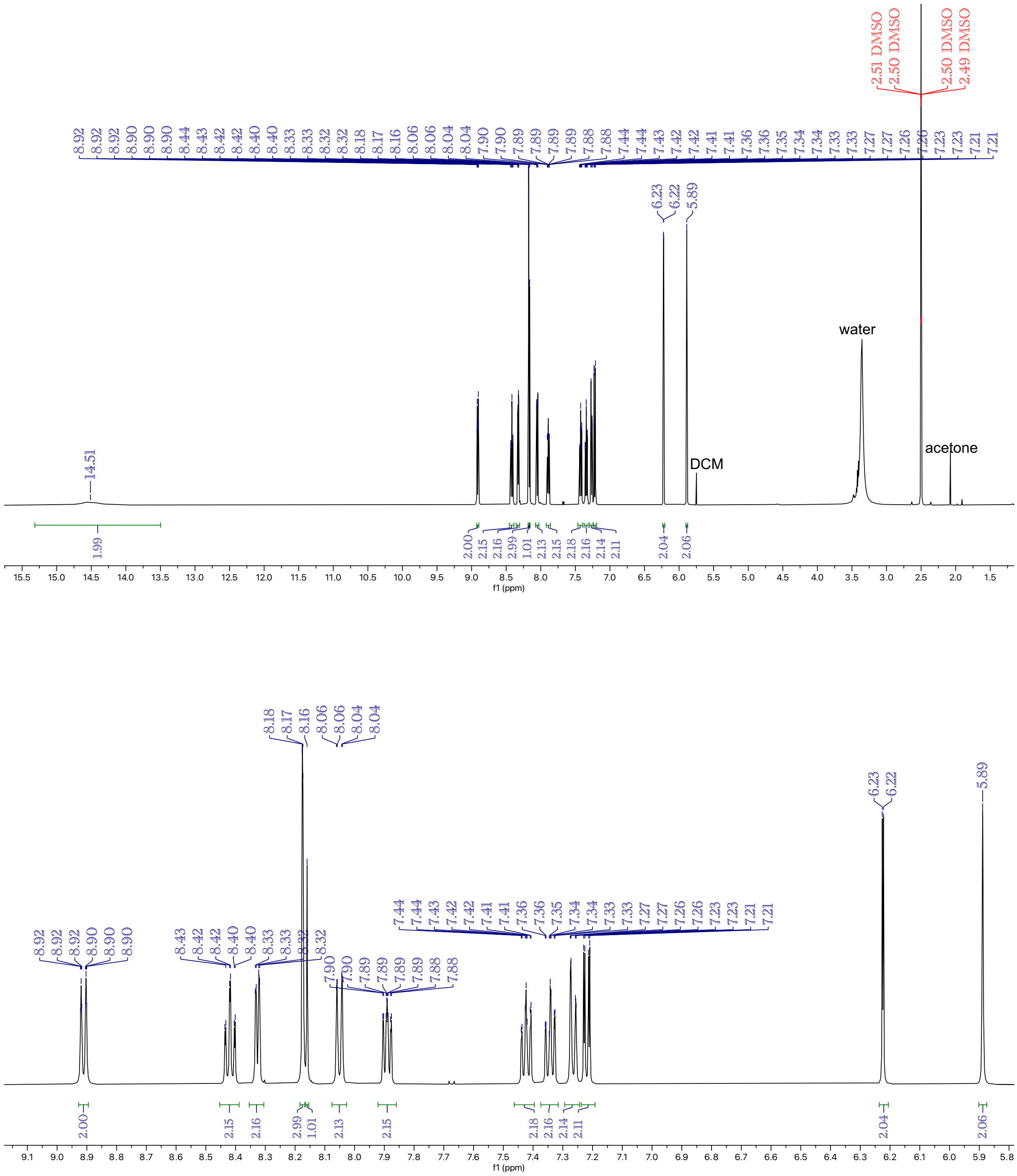

Figure S17. ^{1}H NMR spectrum of [Ir(2-(2-(4-chlorophenyl)-1H-naphtho[2,3-d]imidazole)$_2$(2,2'-bipyridine)][PF_6] (Ir-Cl)

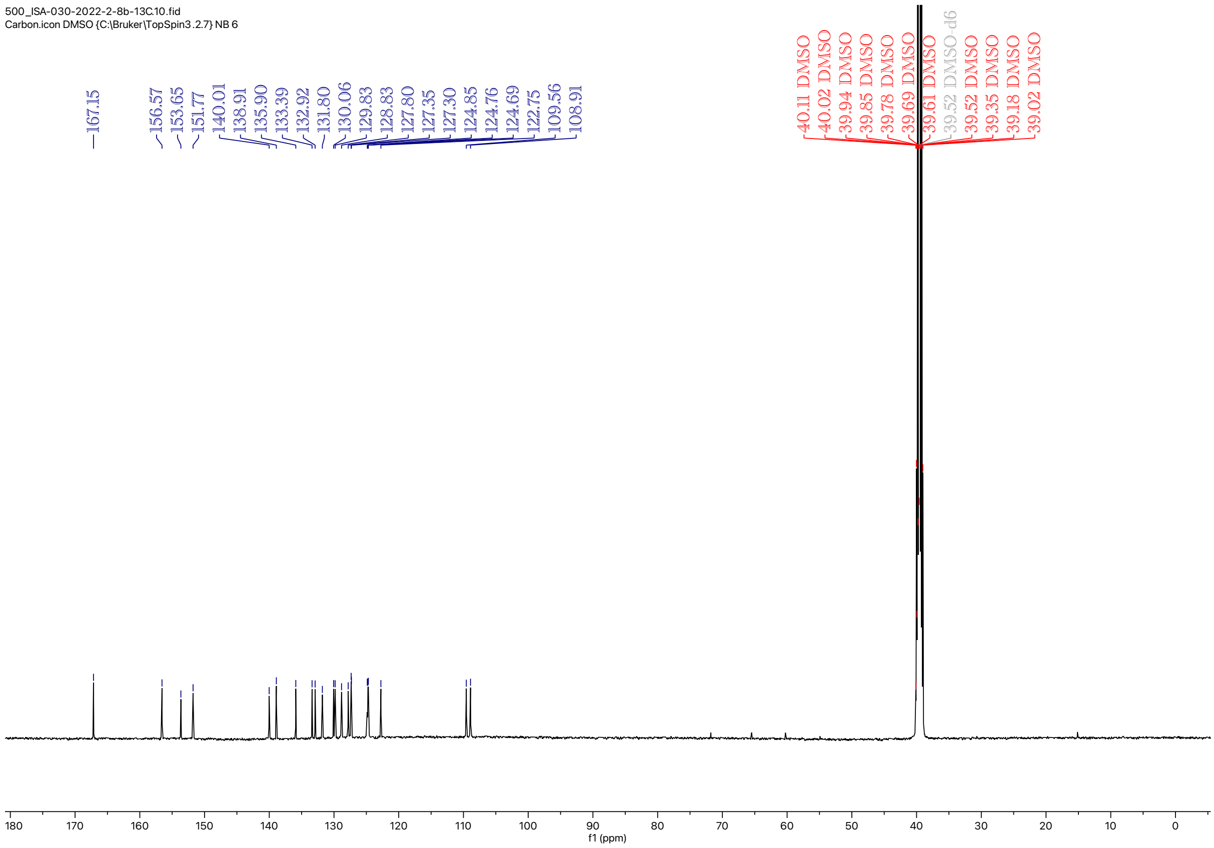

Figure S18. $^{13}C\{^{1}H\}$ NMR spectrum of [Ir(2-(2-(4-chlorophenyl)-1H-naphtho[2,3-d]imidazole)$_2$(2,2'-bipyridine)][PF_6] (Ir-Cl)

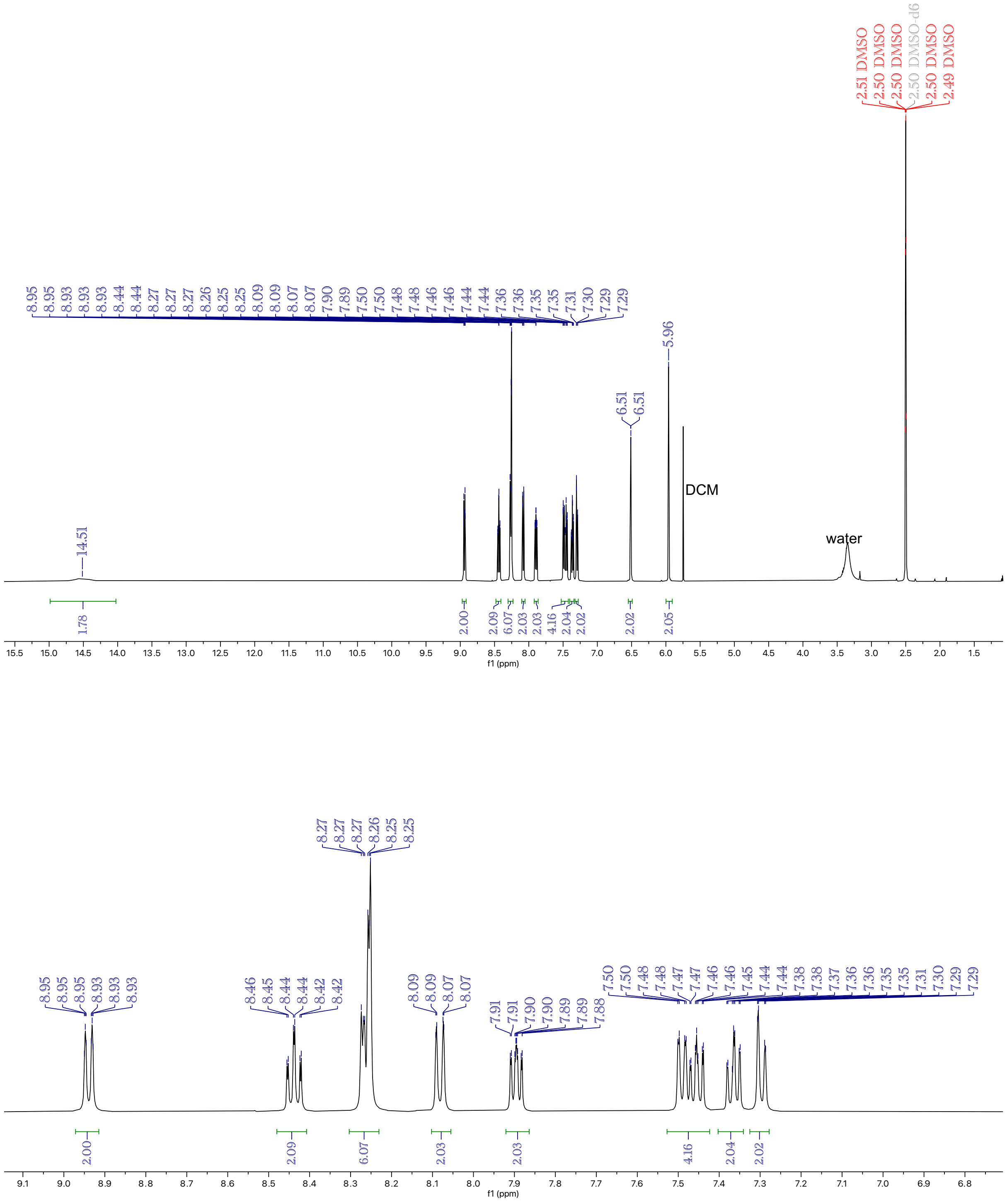

Figure S19. ^{1}H NMR spectrum of [Ir(2-(4-(trifluoromethyl)phenyl)-1H-naphtho[2,3-d]imidazole)$_2$(2,2'-bipyridine)][PF_6] (Ir-CF_3)

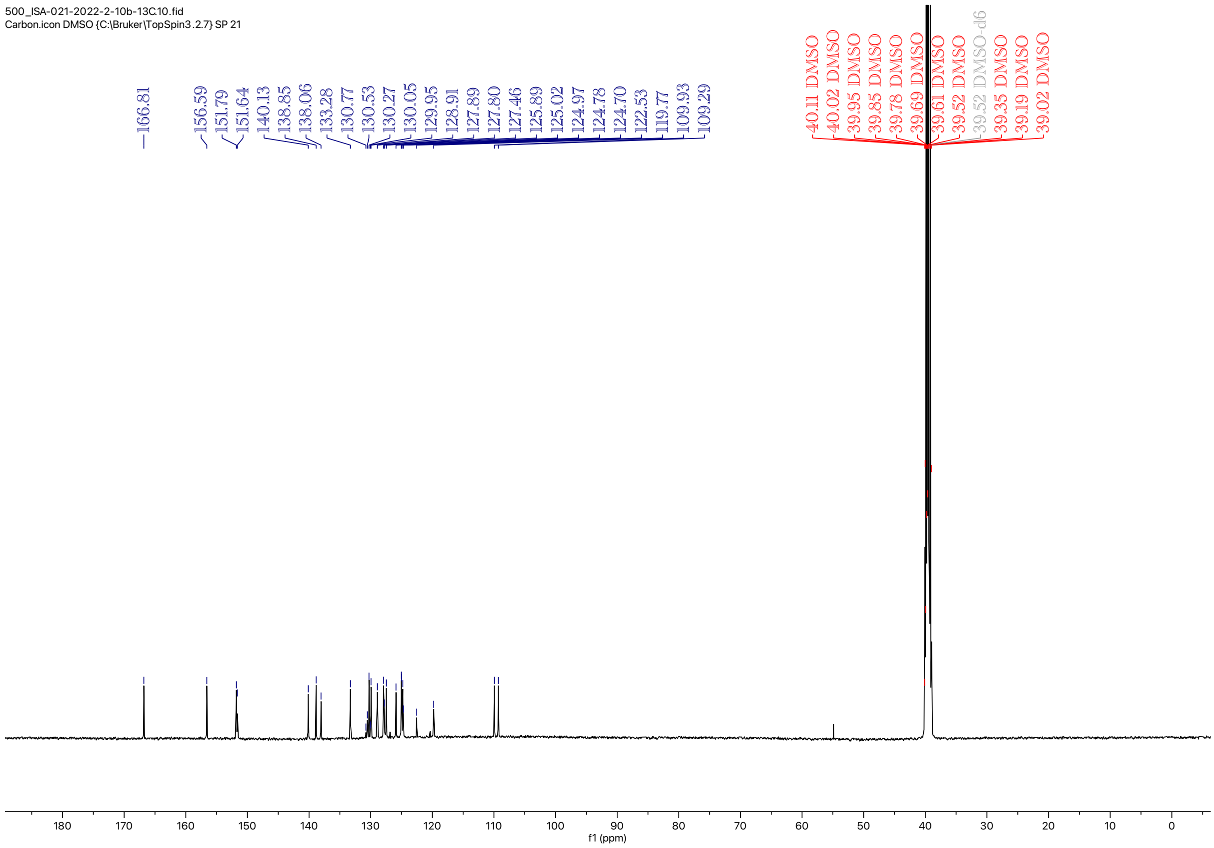

Figure S20. $^{13}C\{^{1}H\}$ NMR spectrum of [Ir(2-(4-(trifluoromethyl)phenyl)-1H-naphtho[2,3-d]imidazole)$_2$(2,2'-bipyridine)][PF$_6$] (Ir-CF$_3$)

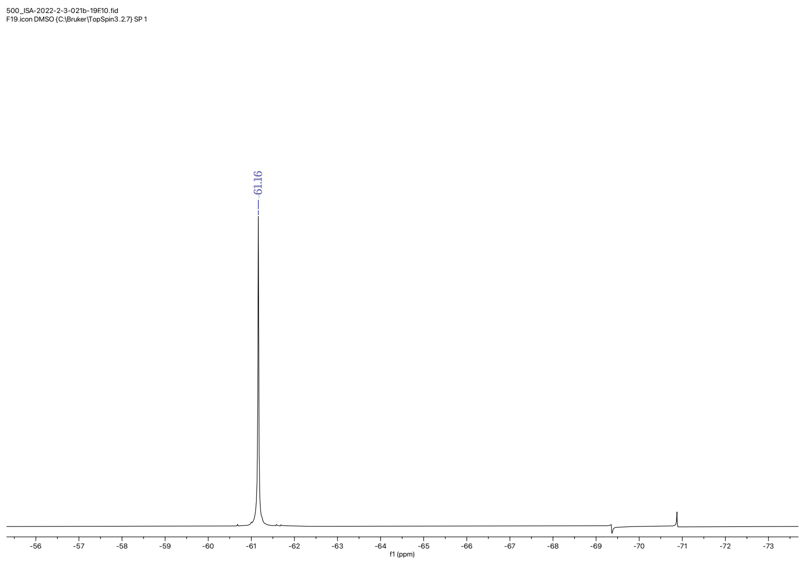

Figure S21. ^{19}F NMR spectrum of [Ir(2-(4-(trifluoromethyl)phenyl)-1H-naphtho[2,3-d]imidazole)$_2$(2,2'-bipyridine)][PF$_6$] (Ir-CF$_3$)

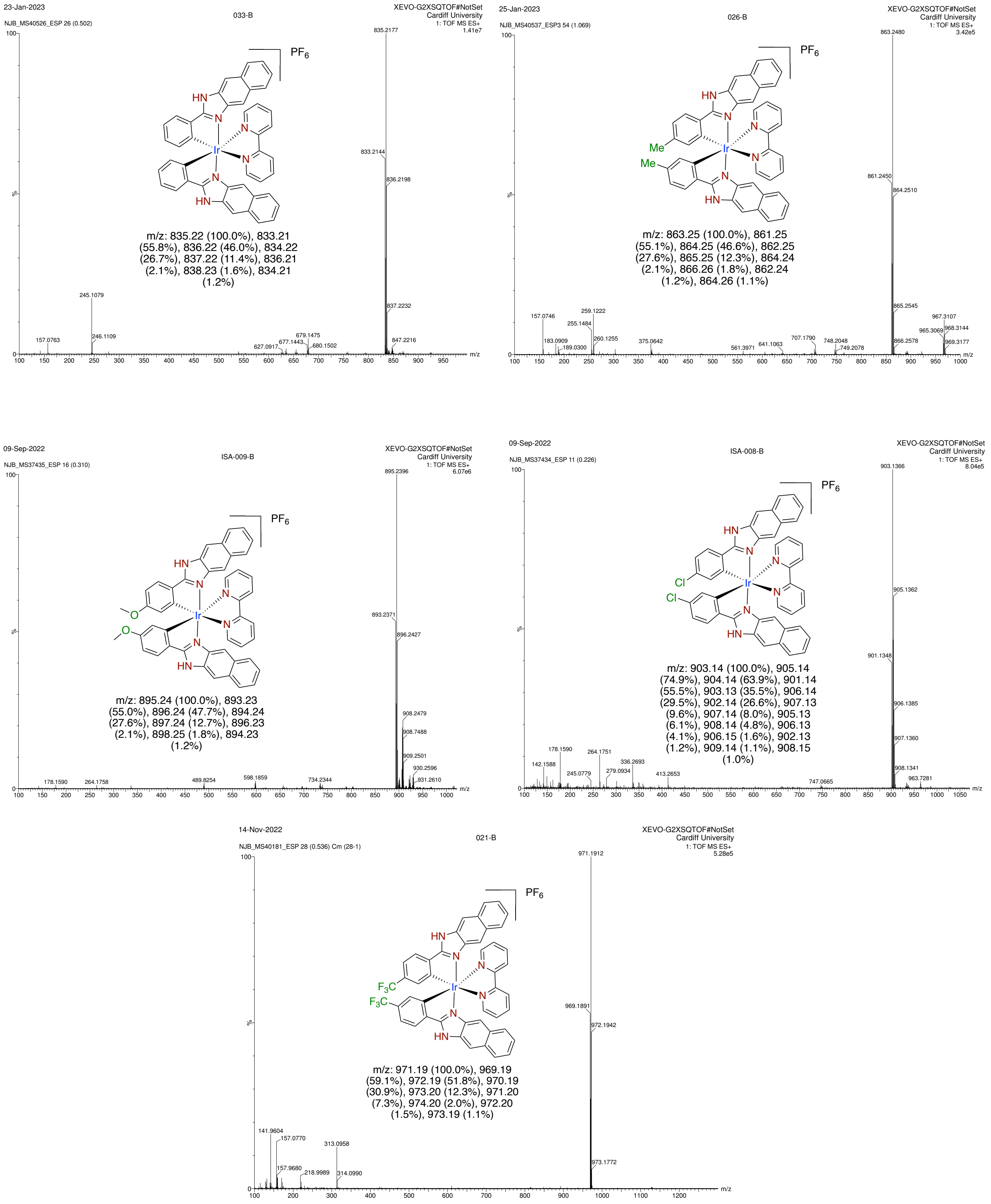

Figure S22. HR MS data for the five Ir(III) complexes.

Table S1. The data collection parameters from the X-ray crystallography.

**Compound**	**Ir-Me**	**Ir-H**	**Ir-OMe**
Formula	$C_{46}H_{34}N_6F_6PIr$	$C_{53}H_{51}ClIrN_6$	$C_{46}H_{34}N_6O_2F_6PIr$
$D_{calc.}$/ g cm^{-3}	1.205	1.416	1.212
μ/mm^{-1}	2.483	2.946	2.424
Formula Weight	1007.96	999.65	1039.96
Colour	orange	red	orange
Shape	block-shaped	plate-shaped	blade-shaped
Size/mm^3	0.280×0.140×0.120	0.260×0.180×0.025	0.360×0.200×0.080
T/K	100(2)	100(2)	100(2)
Crystal System	tetragonal	monoclinic	tetragonal
Space Group	$I4_1/a$	$I2/a$	$I4_1/a$
a/Å	25.1351(4)	21.4244(6)	25.3196(5)
b/Å	25.1351(4)	18.0983(3)	25.3196(5)
c/Å	35.1808(4)	25.9486(6)	35.5697(10)
α/°	90	90	90
β/°	90	111.232(3)	90
γ/°	90	90	90
V/Å^3	22226.3(8)	9378.5(4)	22803.1(11)
Z	16	8	16
Z'	1	1	1
Wavelength/Å	0.71075	0.71075	0.71075
Radiation type	Mo K_α	Mo K_α	Mo K_α
Θ_{min}/°	1.902	1.974	1.887
Θ_{max}/°	28.698	30.509	30.506
Measured Refl's.	175653	162250	144837
Indep't Refl's	14348	14316	17293
Refl's I≥2 σ(I)	7417	10434	7139
R_{int}	0.0476	0.0686	0.0907
Parameters	543	560	830
Restraints	567	0	2295
Largest Peak	2.031	1.666	1.953
Deepest Hole	-1.736	-0.517	-0.704
GooF	1.043	1.033	1.011
wR_2 (all data)	0.2098	0.0703	0.2232
wR_2	0.1736	0.0647	0.1702
R_1 (all data)	0.1247	0.0566	0.1560
R_1	0.0730	0.0347	0.0707

Table S2. Selected bond angles (°) for the three structures.

Ir-Me			
N1	Ir1	N41	99.8(3)
N1	Ir1	N42	89.2(3)
N21	Ir1	N1	171.2(3)
N21	Ir1	N41	86.5(3)
N21	Ir1	N42	98.2(3)
N21	Ir1	C21	79.2(4)
N41	Ir1	N42	77.1(3)
C1	Ir1	N1	79.9(4)
C1	Ir1	N21	94.3(4)
C1	Ir1	N41	176.2(4)
C1	Ir1	N42	99.1(4)
C1	Ir1	C21	87.5(4)
C21	Ir1	N1	93.9(4)
C21	Ir1	N41	96.2(4)
C21	Ir1	N42	173.0(3)

Ir-OMe			
N1	Ir1	N41	98.5(3)
N1	Ir1	N42	88.2(3)
N1	Ir1	N21	170.5(3)
N1	Ir1	N21B	164.1(6)
N41	Ir1	N42	78.2(4)
C1	Ir1	N1	79.8(3)
C1	Ir1	N41	174.1(4)
C1	Ir1	N42	96.1(4)
C1	Ir1	N21	91.9(3)
C1	Ir1	C21	88.1(8)
C1	Ir1	N21B	101.1(7)
C1	Ir1	C21B	90(3)
N21	Ir1	N41	90.2(3)
N21	Ir1	N42	97.4(3)
C21	Ir1	N1	96.3(4)
C21	Ir1	N41	97.7(8)
C21	Ir1	N42	174.3(6)
C21	Ir1	N21	78.6(4)
N21B	Ir1	N41	82.1(7)
N21B	Ir1	N42	107.4(6)
C21B	Ir1	N1	87.7(8)
C21B	Ir1	N41	95(3)
C21B	Ir1	N42	172(2)
C21B	Ir1	N21B	76.4(10)

Ir-H			
N1^{1}	Ir1	N1	172.00(11)
N1	Ir1	N41^{1}	96.05(9)
N1^{1}	Ir1	N41	96.06(9)
N1^{1}	Ir1	N41^{1}	90.20(9)
N1	Ir1	N41	90.20(9)
N41^{1}	Ir1	N41	77.20(12)
C1^{1}	Ir1	N1^{1}	79.31(10)
C1	Ir1	N1	79.31(10)
C1	Ir1	N1^{1}	94.76(10)
C1^{1}	Ir1	N1	94.76(10)
C1^{1}	Ir1	N41	173.97(10)
C1	Ir1	N41^{1}	173.97(10)
C1	Ir1	N41	98.83(9)
C1^{1}	Ir1	N41^{1}	98.83(9)
C1^{1}	Ir1	C1	85.48(14)
N21^{1}	Ir2	N21	170.93(12)
N21^{1}	Ir2	N51	87.40(8)
N21	Ir2	N51^{1}	87.40(8)
N21^{1}	Ir2	N51^{1}	99.74(8)
N21	Ir2	N51	99.74(8)
N51^{1}	Ir2	N51	76.85(12)
C21	Ir2	N21	79.54(10)
C21^{1}	Ir2	N21^{1}	79.54(10)
C21^{1}	Ir2	N21	93.88(10)
C21	Ir2	N21^{1}	93.88(10)
C21^{1}	Ir2	N51^{1}	174.55(10)
C21^{1}	Ir2	N51	97.71(10)
C21	Ir2	N51^{1}	97.71(10)
C21	Ir2	N51	174.55(10)
C21^{1}	Ir2	C21	87.74(15)

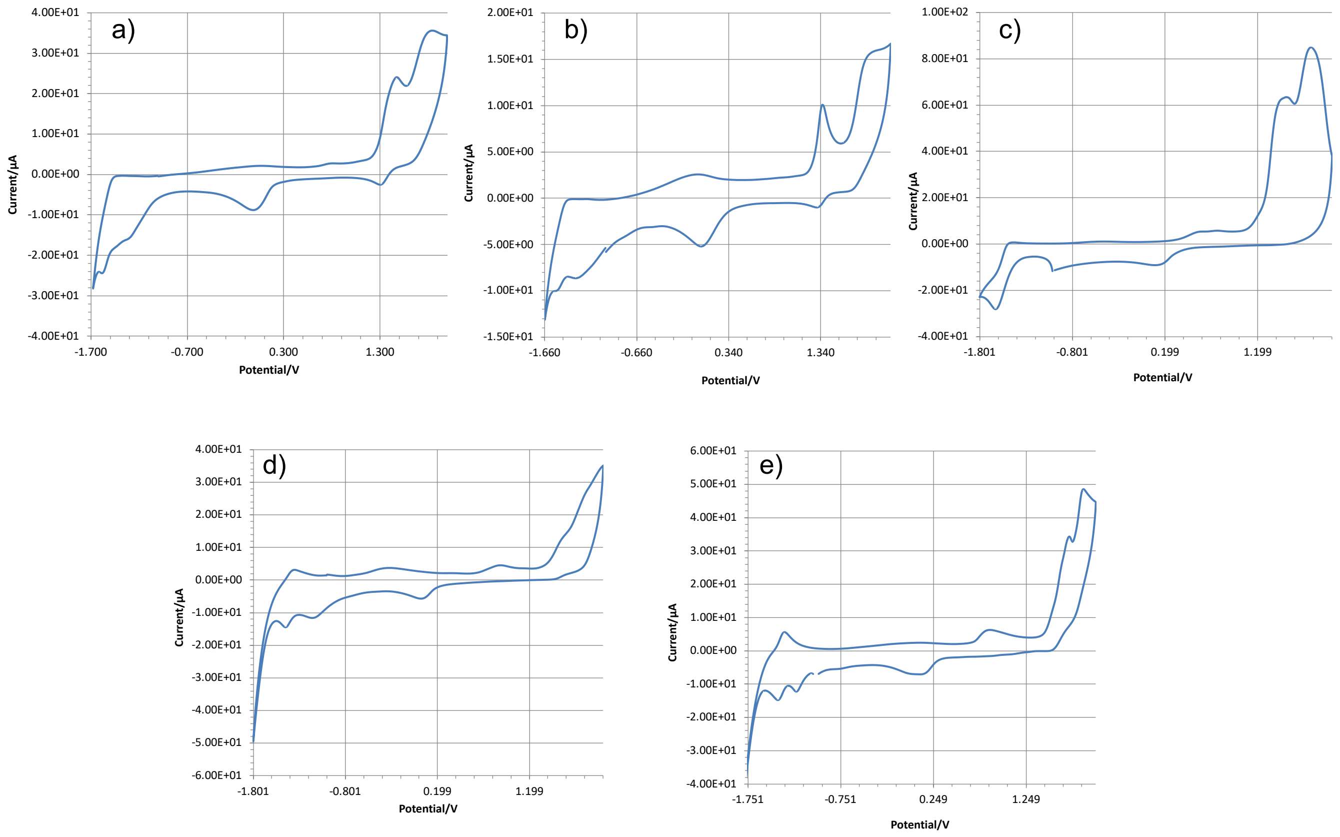

Figure S23. Cyclic voltammetry data for (a) **Ir-H**, (b) **Ir-Me**, (c) **Ir-OMe**, (d) **Ir-Cl** and (e) **Ir-CF**$_3$ complexes in degassed MeCN under different atmospheres (N_2, air).

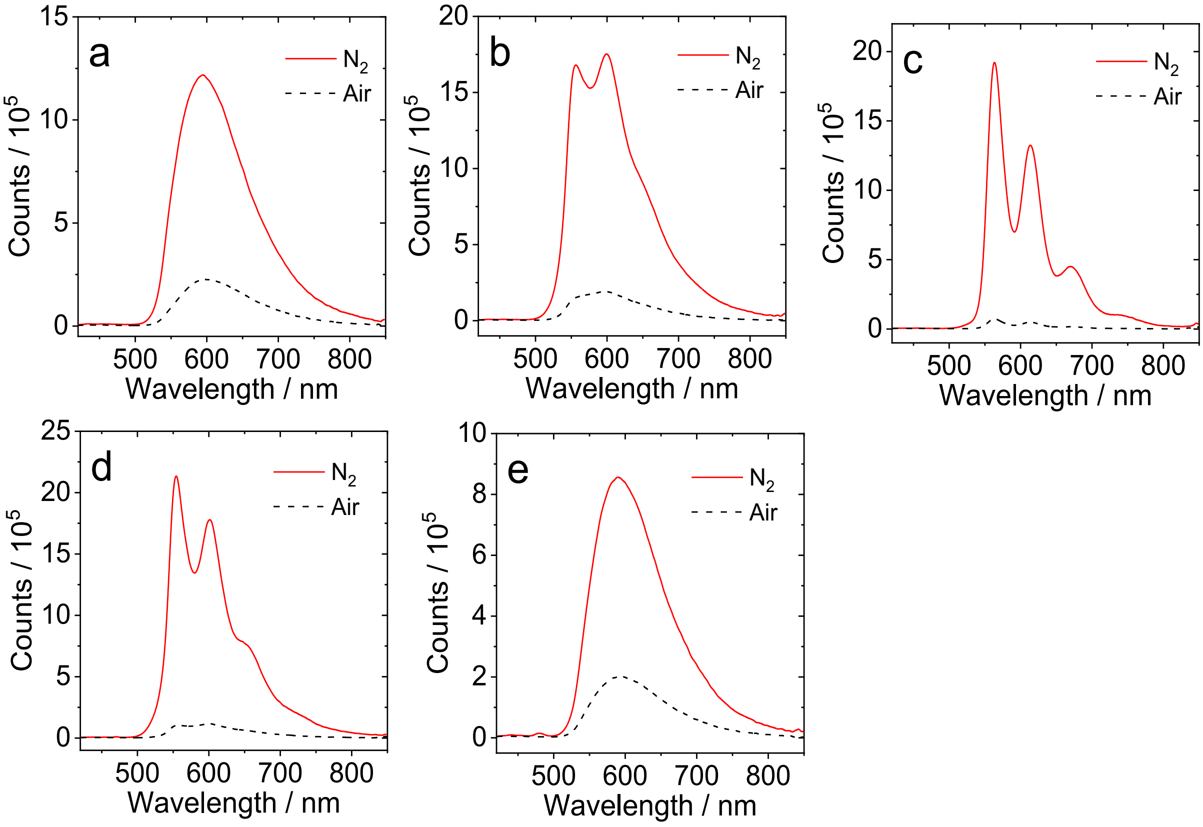

Figure S24. Photoluminescence spectra for (a) **Ir-Me**, (b) **Ir-H**, (c) **Ir-CF$_3$**, (d) **Ir-Cl** and (e) **Ir-OMe** complexes in dichloromethane under different atmospheres (N_2, air). Optically matched solutions were used in each panel (each of the solutions gives the same absorbance at the excitation wavelength, A = 0.100), λ_{ex} = 410 nm, 20 ℃.

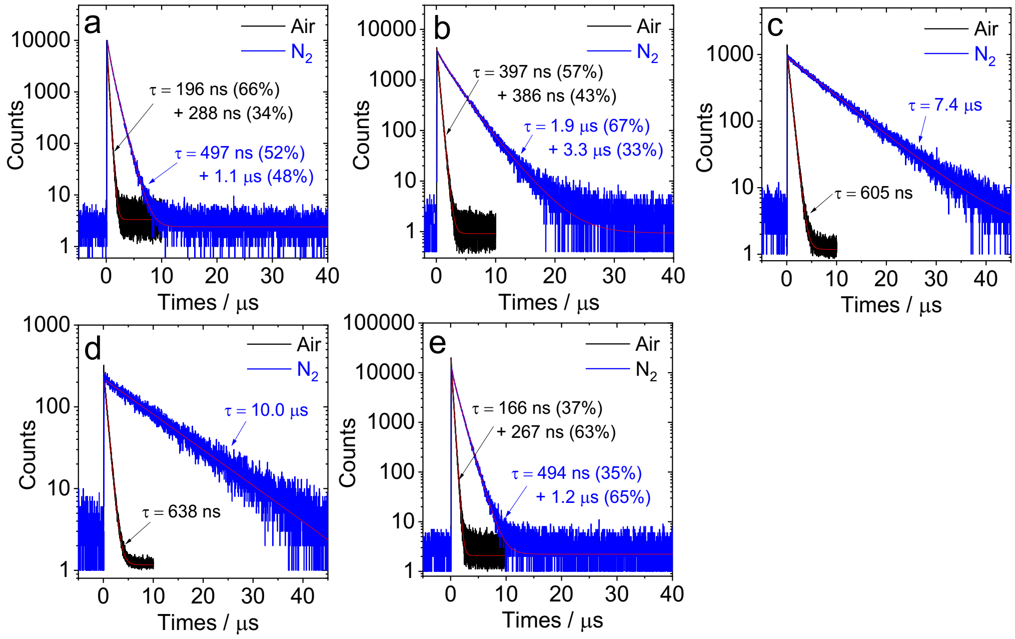

Figure S25. Decay curves of photoluminescence for (a) **Ir-Me**, (b) **Ir-H**, (c) **Ir-CF$_3$**, (d) **Ir-Cl** and (e) **Ir-OMe** complexes in dichloromethane under different atmospheres (N_2, Air). $c = 1.0 \times 10^{-5}$ M, λ_{ex} = 403 nm, 20 ℃.

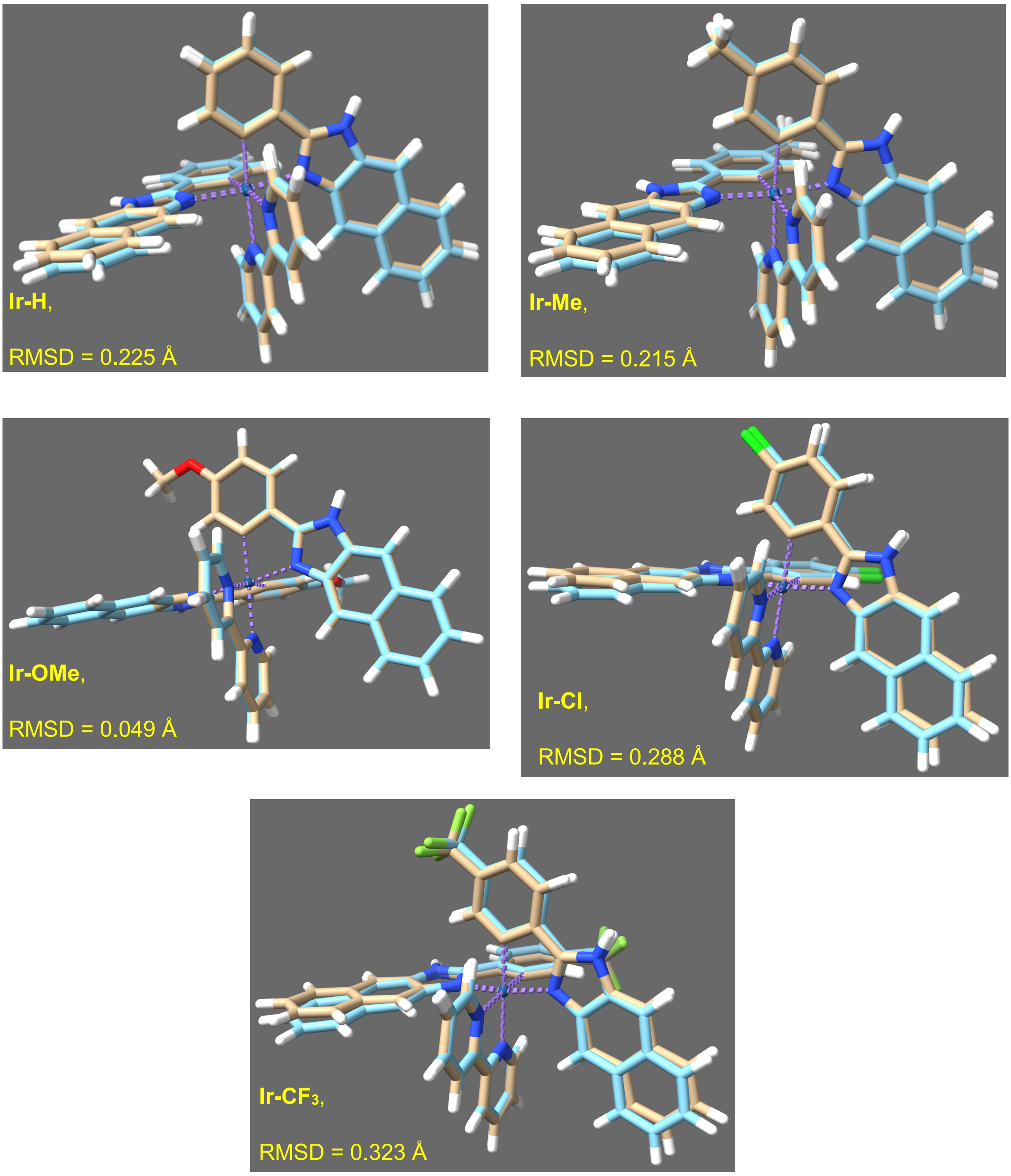

Figure S26. Overlay of the calculated singlet (beige) and triplet (blue) geometries of the complexes.

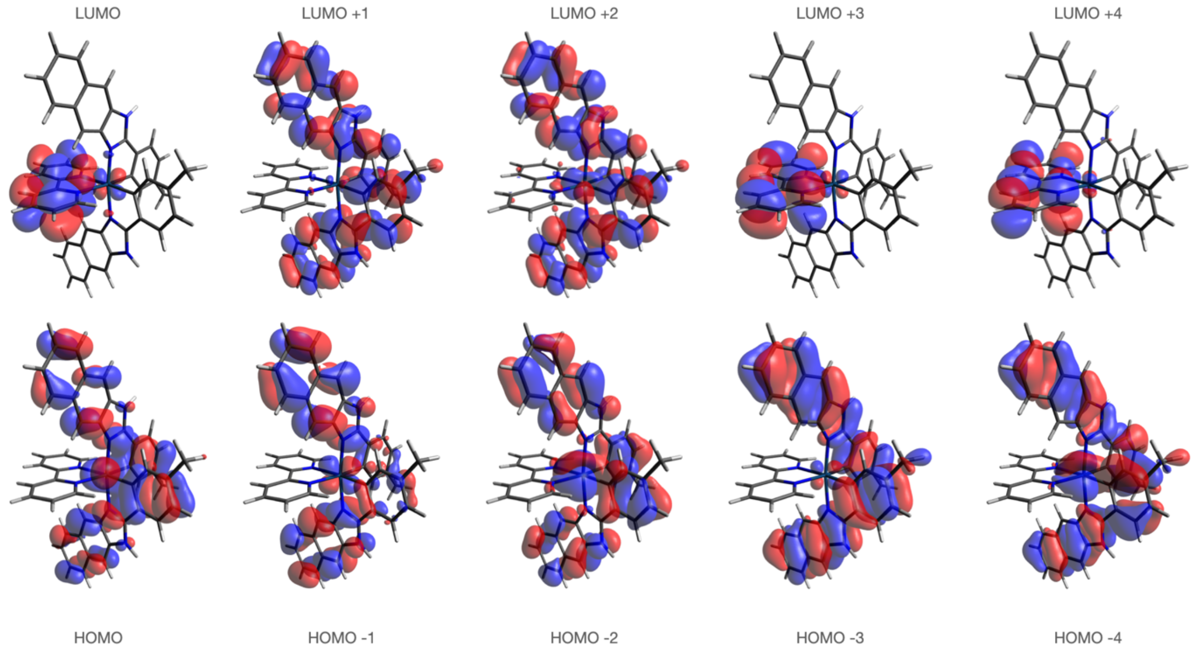

Figure S27. A comparison of the calculated Kohn−Sham frontier molecular orbitals for **Ir-Me**.

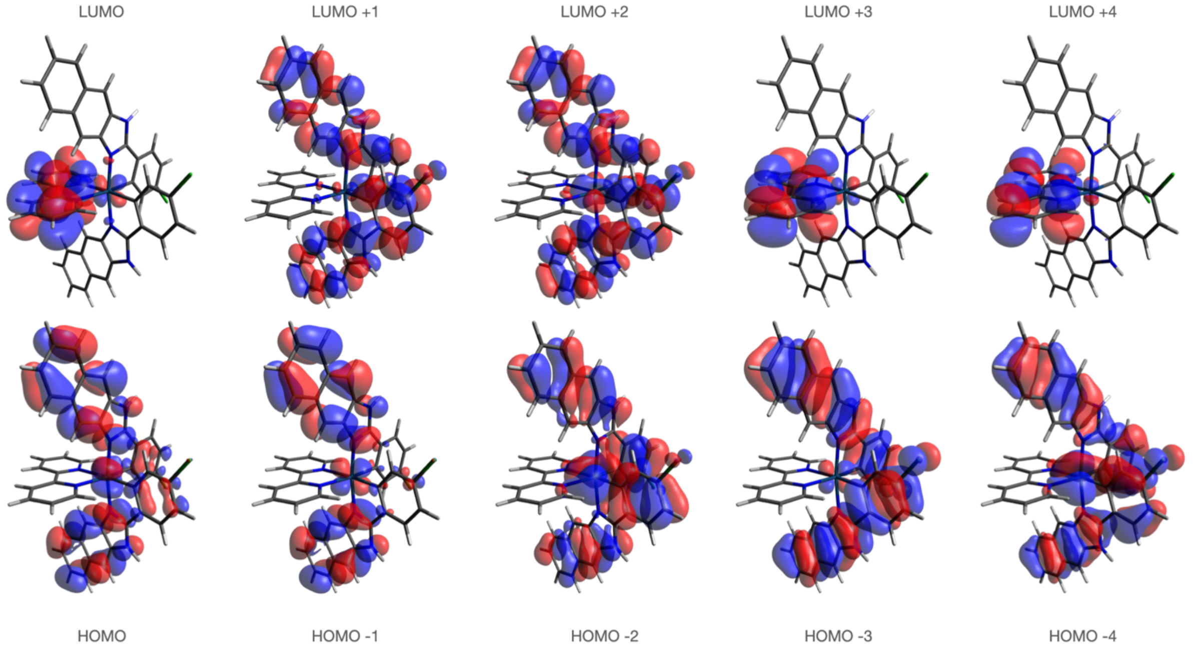

Figure S28. A comparison of the calculated Kohn−Sham frontier molecular orbitals for **Ir-Cl**.

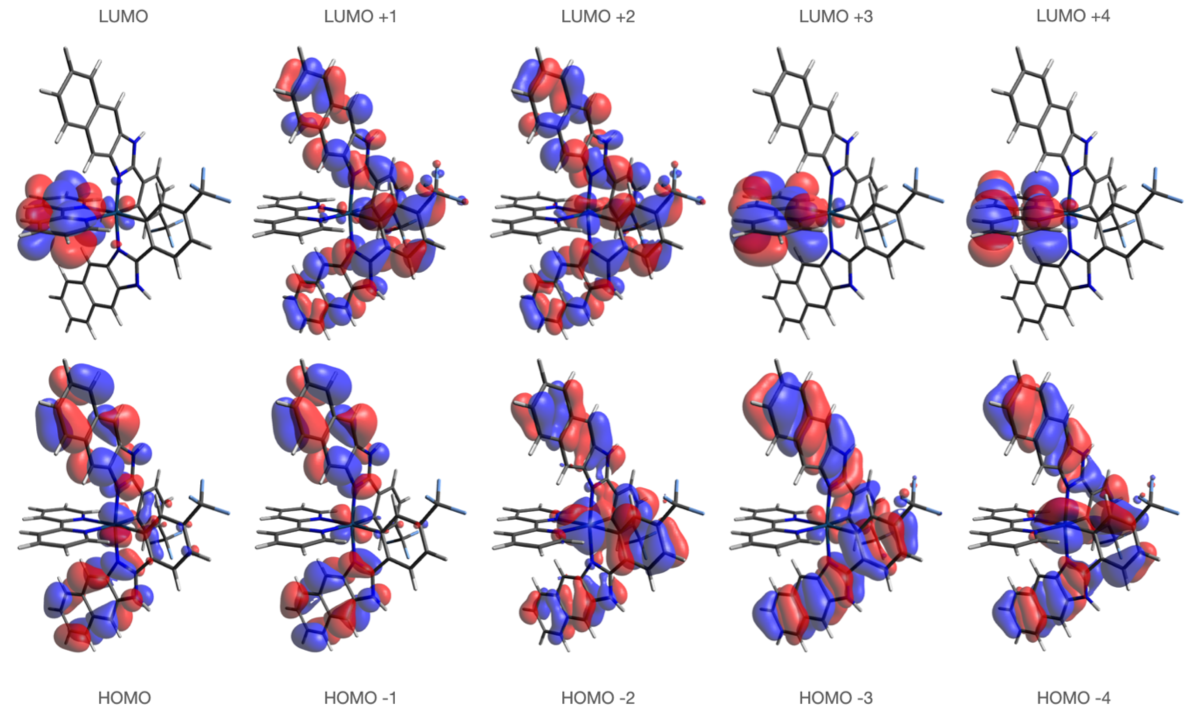

Figure S29. A comparison of the calculated Kohn−Sham frontier molecular orbitals for **Ir-CF$_3$**.

Table S3. Description of the calculated MO contributions, excited states and their associated transitions for **Ir-Me** (L1 and L2 are the cyclometalating ligands; Bpy = 2,2'-bipyridine)

	Moiety Contribution (%)				Orbital Contribution to Excited State	
**Orbital**	**Ir**	**Bpy**	**L1**	**L2**	**Excited State**	**Contributing Transitions (>10%)**
**LUMO +4**	2	96	1	1	1 (354.88 nm, f = 0.0056)	HOMO -2 -> LUMO (18.58%)
**LUMO +3**	3	96	0	0		HOMO -> LUMO (66.17%)
**LUMO +2**	2	2	48	48	2 (336.98 nm, f = 0.5187)	**HOMO -2 -> LUMO +1 (16.15%)**
**LUMO +1**	1	0	49	49		HOMO -1 -> LUMO +2 (14.58%)
						**HOMO -> LUMO +1 (49.28%)**
**LUMO**	3	97	0	0	3 (336.64 nm, f = 0.0289)	HOMO -1 -> LUMO (47.6%)
**HOMO**	23	1	38	38		HOMO -> LUMO +2 (19.67%)
**HOMO -1**	10	1	45	45	4 (329.64 nm, f = 0.2419)	HOMO -1 -> LUMO (34.24%)
**HOMO -2**	23	1	38	38		HOMO -1 -> LUMO +1 (16.14%)
						HOMO -> LUMO +2 (29.6%)
**HOMO -3**	2	0	49	49	5 (326.75 nm, f = 0.0027)	HOMO -6 -> LUMO (13.14%)
**HOMO -4**	16	1	42	42		HOMO -4 -> LUMO (12.44%)
						HOMO -2 -> LUMO (56.11%)
						HOMO -> LUMO (13.85%)

Table S4. Description of the calculated MO contributions, excited states and their associated transitions for **Ir-OMe** (L1 and L2 are the cyclometalating ligands; Bpy = 2,2'-bipyridine)

	Moiety Contribution (%)				Orbital Contribution to Excited State	
**Orbital**	**Ir**	**Bpy**	**L1**	**L2**	**Excited State**	**Contributing Transitions (>10%)**
**LUMO +4**	2	96	1	1	1 (354.84 nm, f = 0.0033)	HOMO -1 -> LUMO (11.46%)
**LUMO +3**	3	96	0	0		HOMO -> LUMO (65%)
**LUMO +2**	2	2	89	8	2 (339.78 nm, f = 0.0126)	HOMO -1 -> LUMO (59.9%)
**LUMO +1**	1	1	8	90		HOMO -> LUMO (11.69%)
**LUMO**	3	97	0	0	3 (334.78 nm, f = 0.6894)	HOMO -1 -> LUMO +1 (13.89%)
**HOMO**	17	1	73	10		**HOMO -> LUMO +1 (15.4%)**
						**HOMO -> LUMO +2 (43.54%)**
**HOMO -1**	11	1	13	76	4 (330.96 nm, f = 0.4195)	HOMO -1 -> LUMO +1 (48.57%)
**HOMO -2**	15	1	59	25		HOMO -> LUMO +2 (19.65%)
**HOMO -3**	0	0	30	70	5 (325.52 nm, f = 0.0021)	HOMO -4 -> LUMO (22.3%)
**HOMO -4**	24	1	37	37		HOMO -2 -> LUMO (56.12%)

Table S5. Description of the calculated MO contributions, excited states and their associated transitions for **Ir-Cl** (L1 and L2 are the cyclometalating ligands; Bpy = 2,2'-bipyridine)

	Moiety Contribution (%)				Orbital Contribution to Excited State	
**Orbital**	**Ir**	**Bpy**	**L1**	**L2**	**Excited State**	**Contributing Transitions (>10%)**
**LUMO +4**	2	97	1	1	1 (339.89 nm, f = 0.0351)	HOMO -2 -> LUMO (26.65%)
**LUMO +3**	3	96	1	1		HOMO -> LUMO (50.13%)
**LUMO +2**	2	2	48	48	2 (334.08 nm, f = 0.422)	HOMO -2 -> LUMO +1 (26.57%)
**LUMO +1**	2	0	49	49		HOMO -1 -> LUMO +2 (16.59%)
						HOMO -> LUMO +1 (32.05%)
**LUMO**	3	97	0	0	3 (332.33 nm, f = 0.1374)	HOMO -2 -> LUMO +2 (15.05%)
**HOMO**	11	0	44	44		HOMO -1 -> LUMO (17.05%)
						HOMO -1 -> LUMO +1 (21.52%)
						HOMO -> LUMO +2 (27.81%)
**HOMO -1**	7	1	46	46	4 (325.93 nm, f = 0.1426)	HOMO -1 -> LUMO (64.63%)
**HOMO -2**	28	1	35	35		
**HOMO -3**	3	0	48	48	5 (318.02 nm, f = 0.0713)	HOMO -4 -> LUMO (14.68%)
**HOMO -4**	21	1	39	39		HOMO -2 -> LUMO (39.6%)
						HOMO -> LUMO (21.61%)

Table S6. Description of the calculated MO contributions, excited states and their associated transitions for **Ir-CF3** (L1 and L2 are the cyclometalating ligands; Bpy = 2,2'-bipyridine)

	Moiety Contribution (%)				Orbital Contribution to Excited State	
**Orbital**	**Ir**	**Bpy**	**L1**	**L2**	**Excited State**	**Contributing Transitions (>10%)**
**LUMO +4**	2	97	1	1	1 (340.28 nm, f = 0.2915)	**HOMO -2 -> LUMO +1 (32.78%)**
**LUMO +3**	3	96	1	1		HOMO -1 -> LUMO +2 (10.83%)
						**HOMO -> LUMO +1 (24.3%)**
**LUMO +2**	2	1	48	48	2 (336.07 nm, f = 0.0654)	HOMO -2 -> LUMO (30.93%)
**LUMO +1**	2	0	49	49		HOMO -> LUMO (38.71%)
**LUMO**	3	97	0	0	3 (335.3 nm, f = 0.1441)	HOMO -2 -> LUMO +2 (20.65%)
**HOMO**	10	0	45	45		HOMO -1 -> LUMO +1 (26.12%)
						HOMO -> LUMO +2 (32.35%)
**HOMO -1**	7	1	46	46	4 (324.02 nm, f = 0.0667)	HOMO -6 -> LUMO (11.8%)
**HOMO -2**	34	2	32	32		HOMO -1 -> LUMO (73.13%)
**HOMO -3**	5	1	47	47	5 (321.52 nm, f = 0.3899)	HOMO -2 -> LUMO +1 (33.65%)
**HOMO -4**	21	1	39	39		HOMO -1 -> LUMO +2 (27%)
						HOMO -> LUMO +1 (19.96%)

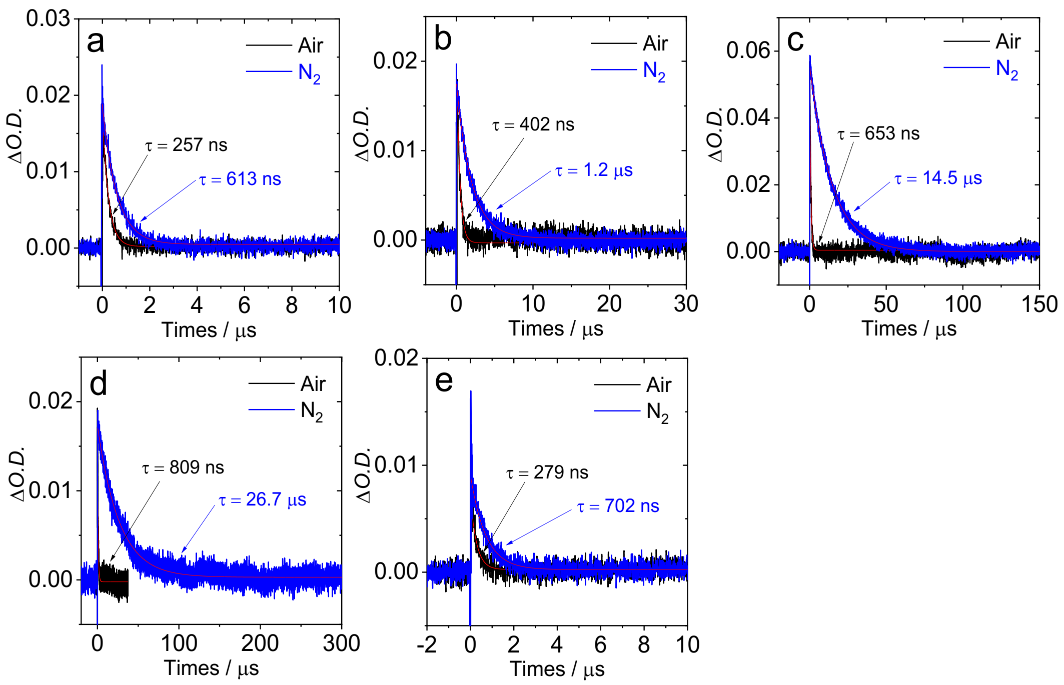

Figure S30. TAS triplet state lifetime for (a) **Ir-Me**, (b) **Ir-H**, (c) **Ir-CF3**, (d) **Ir-Cl** and (e) **Ir-OMe** complexes in dichloromethane under different atmospheres (N_2, Air) upon pulsed laser excitation, λ_{ex} = 420 nm, $c = 3 \times 10^{-5}$ M.

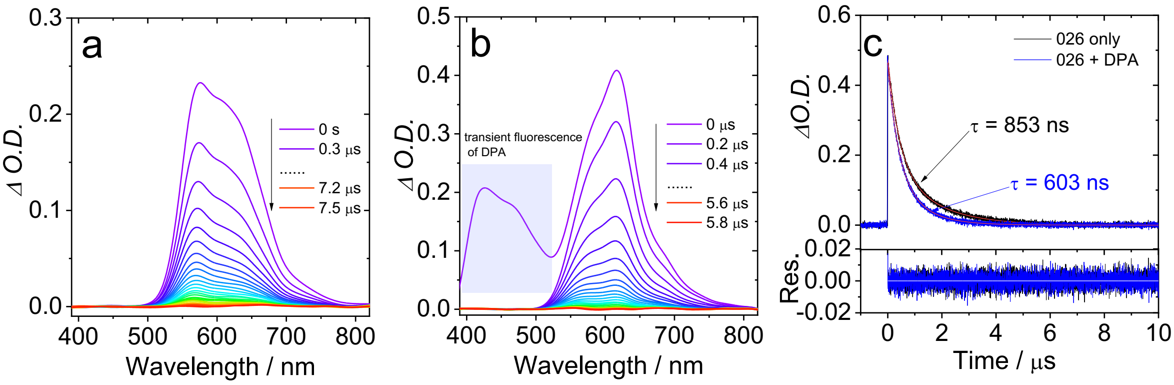

Figure S31. (a) Time-resolved luminescence of **Ir-Me** (c = 3.0 × 10^{-5} M); (b) No delayed fluorescence with **Ir-Me** (c = 3.0 × 10^{-5} M) as the triplet photosensitizer and DPA (c = 1.0 × 10^{-4} M) as the triplet acceptor; (c) The decay traces of the emission at 600 nm, the spike in the delayed fluorescence traces is the scattered laser. In deaerated dichloromethane, 20 °C.

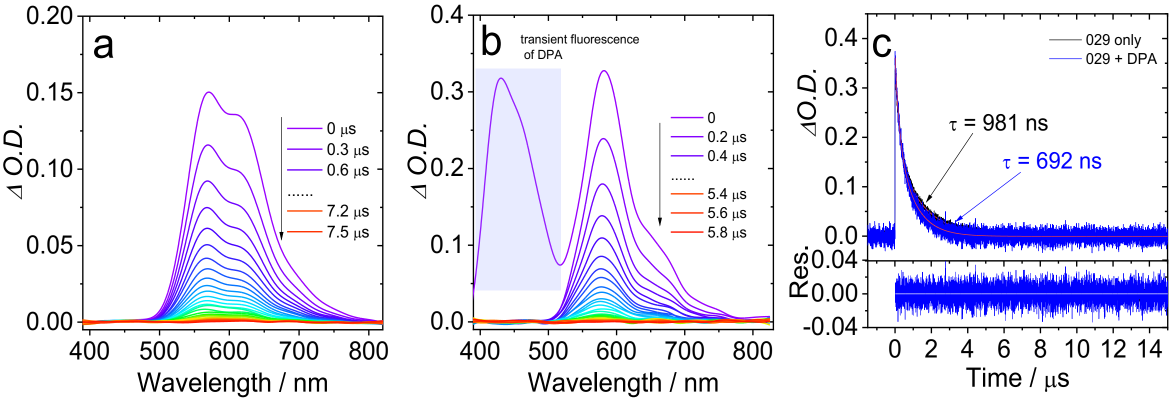

Figure S32. (a) Time-resolved luminescence of **Ir-OMe** (c = 3.0 × 10^{-5} M); (b) No delayed fluorescence with **Ir-OMe** (c = 3.0 × 10^{-5} M) as the triplet photosensitizer and DPA (c = 1.0 × 10^{-4} M) as the triplet acceptor; (c) The decay traces of the emission at 600 nm, the spike in the delayed fluorescence traces is the scattered laser. In deaerated dichloromethane, 20 °C.

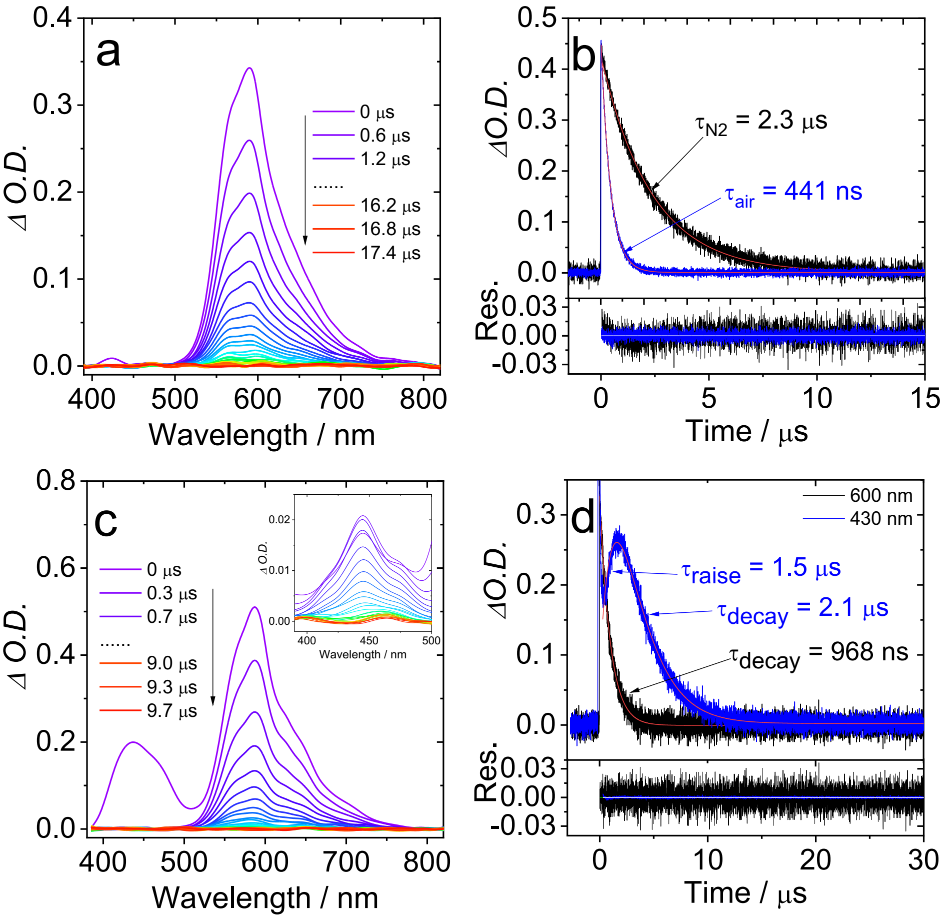

Figure S33. (a) Time-resolved luminescence of **Ir-H** (c = 3.0 × 10^{-5} M). (b) The decay traces of phosphorescence in different atmosphere. (c) Delayed fluorescence with **Ir-H** (c = 3.0 × 10^{-5} M) as the triplet photosensitizer and DPA (c = 1.0 × 10^{-4} M) as the triplet acceptor. (d) The decay traces of the emission at 600 nm ($T_1 \rightarrow S_0$) and 430 nm (^{1}**DPA*** $\rightarrow S_0$), the spike in the delayed fluorescence traces is the scattered laser. In deaerated dichloromethane, 20 °C.

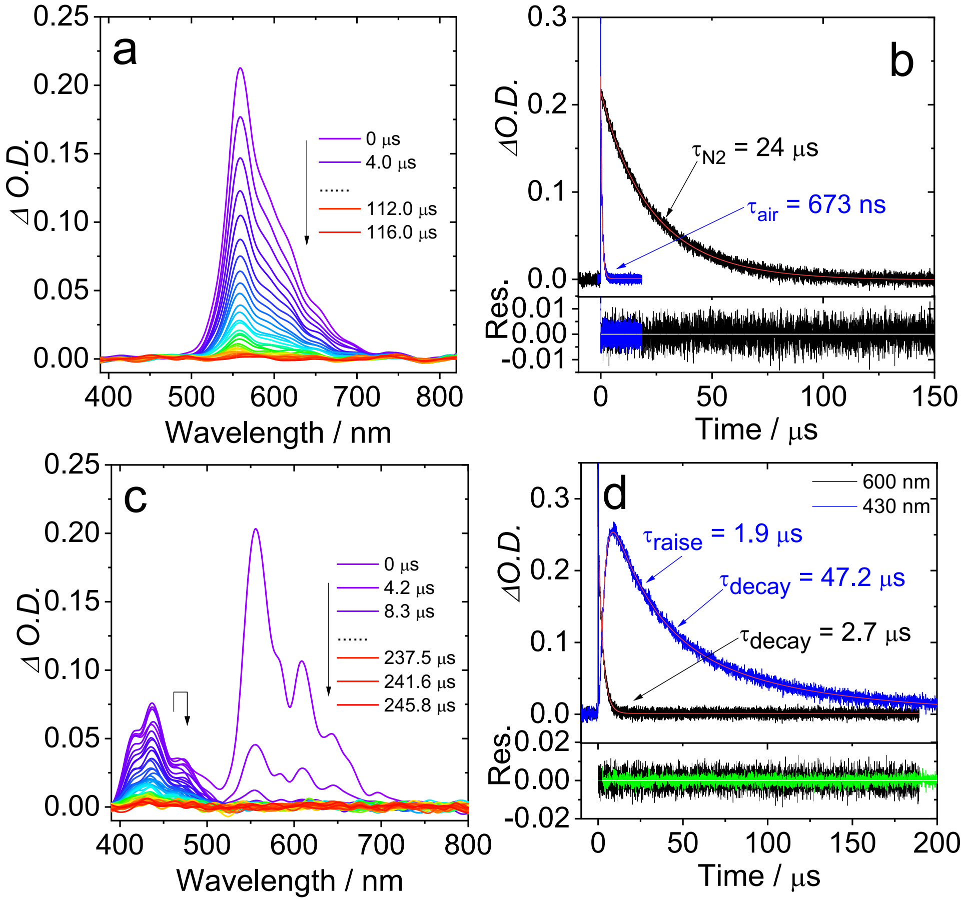

Figure S34. (a) Time-resolved luminescence of **Ir-Cl** (c = 3.0 × 10^{-5} M). (b) The decay traces of phosphorescence in different atmosphere. (c) Delayed fluorescence with **Ir-Cl** (c = 3.0 × 10^{-5} M) as the triplet photosensitizer and DPA (c = 1.0 × 10^{-4} M) as the triplet acceptor. (d) The decay traces of the emission at 600 nm ($T_1 \rightarrow S_0$) and 430 nm (^{1}**DPA*** $\rightarrow S_0$), the spike in the delayed fluorescence traces is the scattered laser. In deaerated dichloromethane, 20 °C.

References

[1] Han, W.-K.; Liu, Y.; Yan, X.; Jiang, Y.; Zhang, J.; Gu, Z.-G. Integrating Light-Harvesting Ruthenium(II)-Based Units into Three-Dimensional Metal Covalent Organic Frameworks for Photocatalytic Hydrogen Evolution. *Angew. Chem. Int. Ed*. **2022**, *61*, e202208791

[2] Wang, Y.; Sarris, K.; Sauer, D. R.; Djuric, S. W. A Simple and Efficient One Step Synthesis of Benzoxazoles and Benzimidazoles from Carboxylic Acids. *Tetrahedron Lett.* **2006**, *47*, 4823.

[3] Gopalaiah, K.; Chandrudu, S. N. Iron(II) Bromide-Catalyzed Oxidative Coupling of Benzylamines with Ortho-Substituted Anilines: Synthesis of 1,3-Benzazoles. *RSC Adv*. **2015**, *5*, 5015

[4] Mobinikhaledi, A.; Deljur, F.; Hamta, A.; Shariatzadeh, S. M. Copper Nitrate Catalyzed Synthesis and Biological Activity Evaluation of Some Naphtho[2,3-d]Imidazoles. *Bulg. Chem. Commun.* **2012**, *44*, 122
